# From Molecular Crystals to Electronic Devices: Investigating Structure–Property Relationships of Iron(III) and Cobalt(II) Coordination Complexes

**DOI:** 10.1021/acs.jpcc.6c04146

**Published:** 2026-09-09

**Authors:** Subhajit Saha, Samit Pramanik, Sudipta Pathak, Riya Sadhukhan, Arnab Ghosh, Dipak K. Goswami, Hon Man Lee, Joaquín Ortega-Castro, Rosa M. Gomila, Antonio Frontera, Subrata Mukhopadhyay

**Affiliations:** † Department of Chemistry, 30167 Jadavpur University, Kolkata 700032, India; ‡ Department of Basic Science and Humanities, Alipurduar Government Engineering and Management College, Bakla 736206, India; § Department of Chemistry, 226429 Haldia Government College, Debhog, Purba Medinipur 721657, West Bengal, India; ∥ Organic Electronics Laboratory, Department of Physics, 30133 Indian Institute of Technology Kharagpur, Kharagpur 721302, India; ⊥ Department of Physics and Natural Science Research Centre of Belda College under Vidyasagar University, Belda 721424, West Bengal, India; # Department of Chemistry, 34910 National Changhua University of Education, Changhua 50058, Taiwan; ¶ Departament de Química, 16745 Universitat de Les Illes Balears, Crta. deValldemossa km 7.5, Palma de Mallorca 07122, (Baleares), Spain

## Abstract

Metal–organic semiconductors based on earth-abundant 3d transition metals offer great potential for optoelectronic devices, yet establishing direct structure–property relationships between specific supramolecular networks, electronic structure, and charge transport remains a major challenge. In this work, we address this gap through a combined experimental and theoretical investigation of two new coordination complexes, [Fe­(**N**_**3**_ **L** **^_1_^**)­(**HN**_**3**_ **L** **^_1_^**)]­Cl_4_.4­(H_2_O) (complex **1**) and [Co­(**HN**_**3**_ **L** **^_1_^**)­(H_2_O)_2_Cl]­Cl_2_.3­(H_2_O) (complex **2**), derived from the previously unexplored 4-imidazole-2,6-di­(pyrazinyl)­pyridine ligand. Single-crystal X-ray diffraction reveals extensive supramolecular architectures stabilized by π···π stacking and hydrogen bonding involving lattice water molecules and chloride counterions. Hirshfeld surface analysis and 2D fingerprint plots quantify the dominant intermolecular contacts, while molecular electrostatic potential, Quantum Theory of Atoms in Molecules, and NCI plot calculations confirm significant anion···π and lone-pair···π interactions in the solid state. Optical absorption studies yield band gaps of 2.02 and 2.16 eV for complexes **1** and **2**, respectively, positioning them for visible-light applications. Fabricated Schottky barrier diodes (ITO/sample/Au) demonstrate rectifying behavior, with device **1** exhibiting a rectification ratio of approximately 66 and a conductivity of 9.2 × 10^–5^ S m^–1^, outperforming device **2** (rectification ratio ∼17, conductivity 1.14 × 10^–5^ S m^–1^). Periodic DFT calculations reveal that the superior electrical performance of complex **1** stems from localized intra-gap states associated with the deprotonated imidazolyl moiety, which facilitate charge transport through hopping mechanisms. These findings elucidate the crucial role of molecular packing and ligand deprotonation in tuning charge transport in functional metal–organic materials.

## Graphical abstract

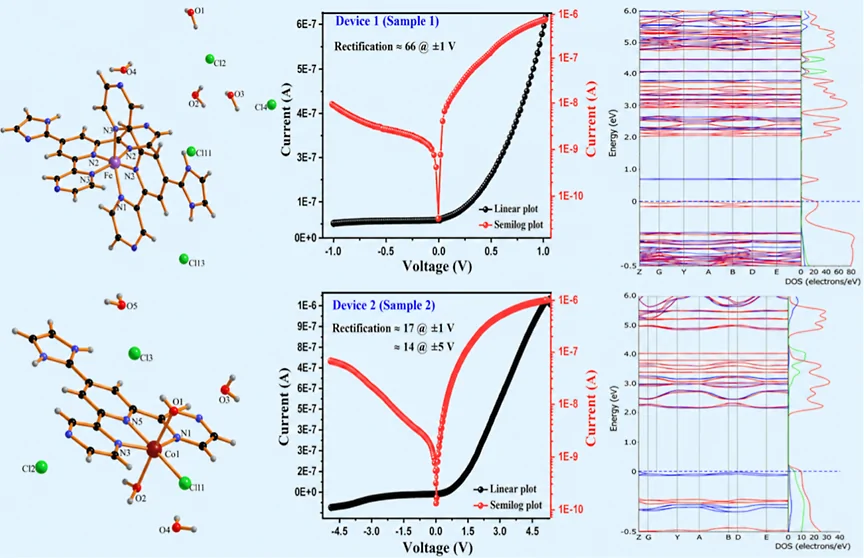

## Introduction

The design and synthesis of metal–organic complexes for optoelectronic applications have emerged as a rapidly expanding field of research due to their distinctive optical and electronic properties originating from metal–ligand interactions.^1–3^ Precise control over the chemical structure at the molecular level enables effective tuning of these properties.^4,5^ When processed as thin films, such materials support the development of low-cost electronic and optoelectronic devices, including LEDs, solar cells, photodetectors, transistors, and chemical or biological sensors.^4,6–12^ Related operational stability principles and defect-passivation strategies have also proven vital in temperature-immune^13^ organic transistor architectures and dielectric energy-storage ceramics.^14^ This overview highlights recent advances in molecular structure control and their extension to solid-state materials for energy conversion and information technology applications.^15,16^

Researchers have widely employed terpyridines and other polypyridines as chelating ligands for d-*block* metal ions because of their strong electron-withdrawing properties and notable coordination chemistry.^17–19^ Terpyridines and polypyridines have emerged as foundational chelating ligands for d-*block* metal ions, primarily due to their robust electron affinity.^20–23^ By utilizing oligo-pyridine motifs, researchers can synthesize exceptionally stable metal complexes that serve as the backbone for various chemical innovations. Furthermore, recent developments in transition-metal-catalyzed multicomponent reactions, such as single-atom copper platforms^24^ or dual Au/Ag systems,^25^ demonstrate the growing utility of tailored metal–nitrogen coordination environments in assembling complex functional architectures. Consequently, terpyridine-based complexes have become a focal point in supramolecular chemistry, with their utility increasingly extending into materials science.^8,26,27^ These complexes exhibit a diverse array of magnetic and optoelectronic properties, making them indispensable for applications such as electrocatalysis and hydrogen evolution.^28^ Beyond these functions, they are also strategically employed as active layers in the fabrication of resistive switching memory devices, as sensitive fluorescent probes for pyrophosphate (PPi) detection, and in the development of advanced photo-functional nanomaterials.^29,30^

Aromatic ligands (such as bipyridine, terpyridine, phenanthroline, porphyrins, or other conjugated systems) are involved in π···π stacking between neighboring molecules that leads to closer packing and better orbital overlap.^31–34^ This enhanced overlap allows partial delocalization of π-electrons across adjacent moieties, facilitating charge transport through the solid.^35^ As a result, metal complexes exhibiting strong π···π interactions often show increased electrical conductivity compared to complexes lacking such interactions.^36,37^ The stacked arrangement creates pathways for electron hopping or band-like charge movement, thereby lowering resistance. Hydrogen bonding similarly promotes close packing and ordered arrangement of neighboring metal complexes, leading to the formation of extended supramolecular networks.^38^ This structural organization facilitates charge transport by providing continuous pathways for the movement of charge carriers.^39^ Hydrogen bonding also stabilizes charge-separated and mixed-valence states in metal complexes, which further promotes metal–ligand charge transfer and improves conductivity.^40^ In addition, hydrogen-bonding interactions also enhance the electrical conductivity of metal complexes by improving molecular organization, stabilizing charge-transfer states, and enabling efficient electron or proton transport pathways.^41^

Iron­(III) and cobalt­(II) metal complexes have emerged as pivotal materials in the design of next-generation optoelectronic devices due to their earth abundance and tunable electronic properties.^42,43^ Unlike traditional noble-metal-based luminophores, iron­(III) complexes utilize ligand-to-metal charge-transfer (LMCT) states to achieve photoactive excited states suitable for light-harvesting applications.^44^ Recent advances have extended the luminescence lifetimes of iron­(III) carbene complexes to the 100 ns range, making them viable candidates for photon upconversion and high-efficiency organic light-emitting diodes.^8^ Simultaneously, cobalt­(II) complexes are extensively researched for their role in dye-sensitized solar cells, where they function as efficient redox mediators that minimize voltage losses.^32^ The structural versatility of cobalt­(II) coordination environments allows for the precise modulation of carrier transport and interfacial polarization in flexible electronics.^45^ Furthermore, these 3d transition-metal complexes are being integrated into hybrid electro-spun nanofibers to create intelligent photodetectors with high specific surface.^46^ The ability to engineer the energy levels of iron­(III) complexes through ligand modification has facilitated their use in photo-redox catalysis, bridging the gap between solar energy conversion and chemical synthesis.^47^ In the realm of stretchable optoelectronics, ionic-conduction-based cobalt systems provide the necessary stability under high operating voltages while mitigating electrochemical degradation.^48^ Collectively, these developments demonstrate that the shift from scarce 4d/5d metals to iron- and cobalt-based systems is essential for the sustainable commercialization of third-generation photovoltaics. By optimizing doublet-triplet energy transfer in iron­(III) systems and enhancing the redox kinetics in cobalt­(II) platforms, researchers are achieving performance levels previously reserved for iridium and ruthenium analogues.^49–51^

Despite these advances, a fundamental research gap remains regarding how noncovalent network assemblies (such as π···π stacking, hydrogen-bonding, and anion/lone-pair···π interactions) coupled with ligand protonation states directly dictate solid-state intra-gap electronic states and charge transport mechanisms in 3d transition-metal complexes. In this work, we combine experimental and theoretical investigations of two new iron­(III) and cobalt­(II) coordination complexes derived from 4-imidazole-2,6-di­(pyrazinyl)­pyridine which has not been explored to date. We provide a detailed analysis of their supramolecular architectures, which are stabilized by extensive networks of π···π and hydrogen-bonding interactions for the title complexes **1** and **2**. The Hirshfeld surface (HS) analysis, together with the corresponding two-dimensional fingerprint plots, provides a comparative insight into the intermolecular interactions, highlighting both their common features and their distinct contributions to the overall supramolecular architectures. To complement the experimental findings, a theoretical investigation was performed for complex **2**, owing to its more accessible molecular size and electronic configuration, using molecular electrostatic potential (MEP) surface analysis, Quantum Theory of Atoms in Molecules (QTAIM), and NCI plot to quantify the nature of the supramolecular assembly and the impact of anion···π and lone-pair···π interactions. Furthermore, the potential of these materials in electronic applications was evaluated through the fabrication of ITO/sample/Au Schottky devices, supported by periodic DFT calculations, to elucidate the charge transport mechanisms and the role of localized intra-gap states in enhancing electrical conductivity. Therefore, this work provides new insights into the development of previously unexplored Fe­(III) and Co­(II) coordination complexes of 4-imidazole-2,6-di­(pyrazinyl)­pyridine and the establishment of a direct structure–property relationship between supramolecular interactions, ligand-centered intra-gap states, and charge transport behavior through combined experimental device fabrication and periodic DFT analysis.

## Experimental Section

### Materials and Apparatus

All chemical reagents of analytical grade and solvents of spectroscopic grade were purchased from commercial suppliers and employed without further purification. Doubly distilled water prepared freshly was utilized throughout the synthetic process, and all reactions were conducted under aerobic conditions. Elemental analyses (carbon, hydrogen, and nitrogen) were conducted using a PerkinElmer 2400 Series-II CHN analyzer, USA. Fourier transform infrared (FTIR) spectra were captured using a Perkin Elmer LX-1 FT-IR spectrophotometer over the range of 4000 to 400 cm^–1^, utilizing a contemporary diamond attenuated total reflectance accessory. Keithley 2450 source-measure units were used for the electrical characterizations of the Schottky devices at room temperature (300 K) under dark conditions. Avaspec-3648 UV–Vis–NIR spectrophotometer was engaged to record the ground-state absorption spectra of the concerned complexes (spin-coated thin films on ITO/glass substrates).

### Synthesis of 4-Imidazole-2,6-di­(pyrazinyl)­pyridine [N_3_L**^_1_^**]

The ligand **N**_**3**_ **L**_**1**_was synthesized by using acetyl pyrazine and 2-imidazolecarboxaldehyde in the presence of an aqueous NH_3_ (35%) solution, according to the following literature method.^52^ Main FTIR absorptions, (KBr, cm^–1^): 3280 (br s), 1610 (vs), 1546 (vs), 1519 (s), 1461 (s), 1368 (s), 1319 (s), 1113 (s), 1018 (vs), 850 (vs), 719 (s), 705 (vs) (Figure S1, Supporting Information).

### Synthesis of [Fe­(N_3_L**^_1_^**)­(HN_3_L**^_1_^**)]­Cl_4_ (Complex **1**)

An ethanolic solution (5 mL) of anhydrous FeCl_3_ (0.162 g, 1.0 mmol) was added to a suspension (20 mL) of the ligand, **N**_**3**_ **L** **^1^** (0.301 g, 1.0 mmol), in ethanol with constant stirring for 2 h (Scheme S1, Supporting Information) at 65 °C. A wine-red-colored solution was obtained after 2 h of complete addition. The solution was then settled for 14 h. The resultant bluish-violet-colored precipitate was filtered, and the filtrate was kept undisturbed for crystallization. After 2 weeks, violet X-ray quality crystals of complex **1** were obtained from the mother liquor (yield: 39%). Anal. calculated for C_32_H_31_N_14_FeO_4_Cl_4_: C, 43.99; H: 3.55; N: 22.45%. Found: C, 43.98; H, 3.54; N: 22.45%. Main FTIR absorptions, (KBr, cm^–1^): 3353 (br s), 2923 (br s), 2555 (br s), 1644 (vs), 1601 (vs), 1478 (vs), 1403 (vs), 1370 (s), 1295 (vs), 1143 (vs), 1091 (vs), 1039 (vs), 1020 (vs), 907 (vs), 850 (vs), 756 (vs) (Figure S2, Supporting Information).

### Synthesis of [Co­(HN_3_L**^_1_^**)­(H_2_O)_2_Cl]­Cl_2_ (Complex **2**)

An aqueous solution (3 mL) of CoCl_2_.6H_2_O (0.237 g, 1.0 mmol) was added dropwise to an aqueous suspension (15 mL) of the ligand, **N**_**3**_ **L** **^_1_^** (0.301 g, 1.0 mmol). After 5 min of stirring, 3 drops of concentrated HCl were added dropwise to make a solution having pH = 1. Within few seconds after the addition of conc. HCl, complete solution color turns into deep red. This solution was then stirred for 2 h, filtered, and kept undisturbed for crystallization. After five months, red-colored X-ray quality crystals of complex **2** were obtained from the mother liquor (yield: 61%). Anal. calculated forC_16_H_22_N_7_CoO_5_Cl_3_: C, 34.44; H, 3.94; N, 17.58%. Found: C, 34.44; H, 3.93; N, 17.56%. Main FTIR absorptions, (KBr, cm^–1^): 3353 (br s), 2735 (br s), 1610 (s), 1459 (vs), 1393 (vs), 1299 (vs), 1190 (vs), 1143 (s), 1091 (s), 1025 (s), 864 (s), 761 (s) (Figure S3, Supporting Information).

Details of X-ray crystallographic analysis, theoretical methods, and device fabrication have been provided in the Supporting Information.

## Results and Discussion

### FT-IR Analysis

The characteristic IR stretching frequencies for O–H, N–H, and C–H vibrations are observed in the ranges of 3300–3600 cm^–1^ (broad), 3100–3500 cm^–1^ (broad), and 2700–3100 cm^–1^, respectively. In the IR spectra of complexes **1** and **2**, a broad absorption band accompanied by several shoulder peaks is observed in the 3000–3600 cm^–1^ region. These bands are attributed to the overlapping O–H and N–H stretching vibrations, indicating the presence of hydrogen-bonded hydroxyl, coordinated and noncoordinated water molecules, and/or imino group in the complexes. In infrared (IR) spectroscopy, the C=C stretching vibrations of aromatic rings and the C=N stretching vibrations of heteroaromatic or azomethine groups are generally observed in the 1650–1600 cm^–1^ region. Owing to the higher bond strength and force constant of the C=N bond compared with the C=C bond, the C=N stretching vibration typically appears at a slightly higher wavenumber. In the IR spectrum of complex **1**, the absorption bands at 1644 and 1601 cm^–1^ are assigned to the C=N and aromatic C=C stretching vibrations, respectively. Similarly, the corresponding bands appear at 1648 and 1610 cm^–1^ in the IR spectrum of complex **2**, confirming the presence of C=N and aromatic C=C functionalities in the coordinated ligands. IR peaks in the 1500–1400 cm^–1^ region are generally attributed to the skeletal stretching vibrations of aromatic and heteroaromatic compounds (mixed C=C/azomethine (C=N) skeletal modes). This region may also contain contributions from C–H in-plane bending, CH_2_ scissoring, and CH_3_ asymmetric and symmetric bending (umbrella deformation) vibrations, depending on the ligand structures of the complexes. Accordingly, several absorption bands are observed for both complexes in the 1500–1400 cm^–1^ region, which are primarily assigned to the aromatic ring skeletal (C=C and C=N) vibrations, with possible contributions from C–H bending. These functional groups and their corresponding absorption bands are also present in the free ligand; however, slight shifts in their positions are observed upon complex formation. These shifts indicate the coordination of the ligand to the metal center, resulting in changes in the electronic environment of the corresponding functional groups.

### Structural Description for Complexes **1** and **2**

The molecular structures of complex **1** and complex **2** along with the selected atom numbering scheme are presented in Figures 1 and 2, respectively. Here, we have used Diamond version 3.1 ^53^ to generate the figures of the X-ray structures for both the complexes. Structural analysis reveals that both the complexes crystallize in a monoclinic system with the space group *P*2_1_/*c*, and their unit cell comprises four complex units (Table S1). Bond lengths (Å) and bond angles (°) of the complex **1** and complex **2** are shown in Tables S2 and S3, respectively (Supporting Information). Complex **1** contains a general formula [Fe­(**N**_**3**_ **L^_1_^**)­(**HN**_**3**_ **L^_1_^**)]­Cl_4_.4­(H_2_O), whereas the general formula of complex **2** is [Co­(**HN**_**3**_ **L^_1_^**)­(H_2_O)_2_Cl]­Cl_2_.3­(H_2_O). In both of the cases, **N**_**3**_ **L^_1_^** [4-imidazole-2,6-di­(pyrazinyl)­pyridine] acts as a tridentate chelating ligand.

**Figure 1:**
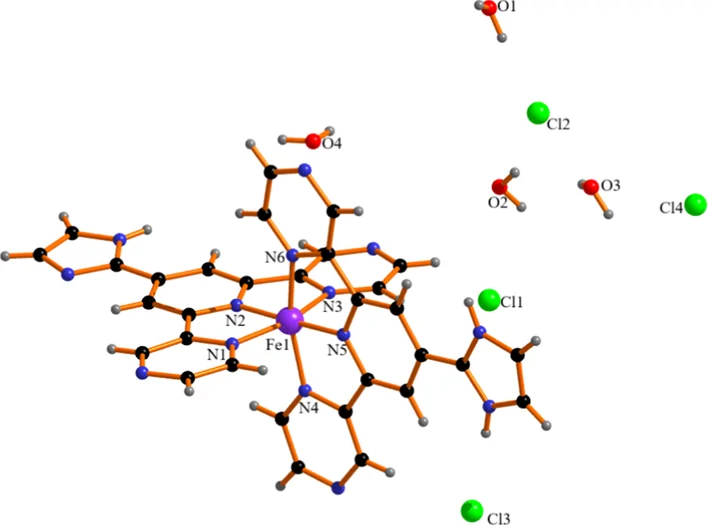
Molecular structure of complex **1**.

**Figure 2:**
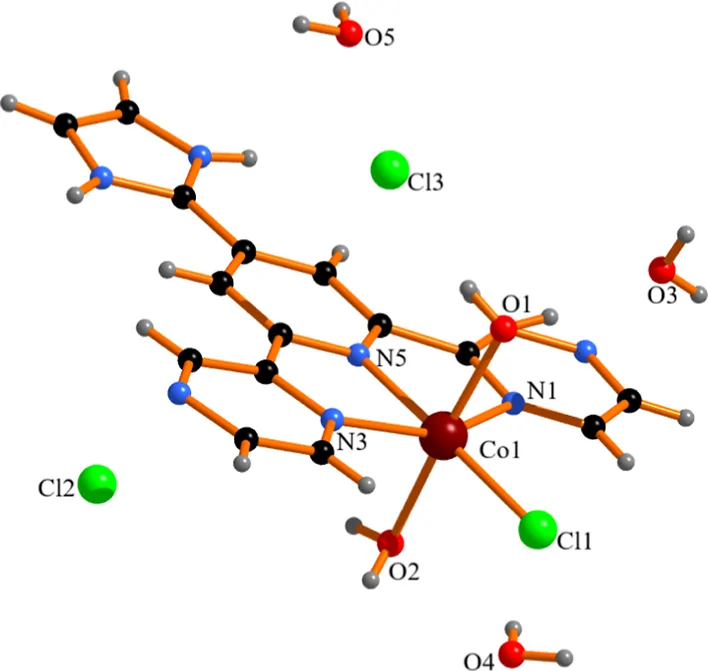
Molecular structure of complex **2**.

The variation in the number of coordinated tridentate ligands (**N**_**3**_ **L**_**1**_) with different metal ions in complexes **1** and **2** not only leads to distinct structural architectures but also significantly influences the extent of their supramolecular behavior. The coordination mode around the metal center can be best described as the meridional geometry of the distorted octahedron for complex **1**. For complex **1**, the two pyrazinyl nitrogen atoms (N2 and N5) and the two pyridyl nitrogen atoms (N4 and N6) of our title ligand (**N**_**3**_ **L^_1_^**) generate the basal plane, while the axial positions are occupied by another two pyrazinyl nitrogen atoms (N1 and N3). The average Fe–N_eq_ bond distance is 1.935 Å [Fe1–N2 = 1.888(2) Å, Fe1–N4 = 1.977(2) Å, Fe1–N5 = 1.886(2) Å, and Fe1–N6 = 1.990(2) Å]. The axial nitrogen atoms are positioned somewhere at a longer distance [Fe1–N1 = 1.998(3) Å and Fe1–N3 = 1.977(2) Å] than the equatorial bond distances [Fe1–N_eq_ = 1.935 Å]. For complex **2**, the basal plane is formed by the two oxygen atoms (O1 and O2) of two water molecules and the one pyrazinyl nitrogen atom (N5) of the title ligand (**N**_**3**_ **L^_1_^**) and one chloride ion (Cl1). The trans-axial positions are occupied by the two pyrazinyl nitrogen atoms (N1 and N3) of the title ligand (**N**_**3**_ **L^_1_^**). In the basal plane, the Co1–N5 bond length is 2.075(2) Å, Co1–Cl1 is 2.3455(10) Å, whereas the axial Co–O bond lengths [2.100(2) Å and 2.120(2) Å] are shorter compared to the equatorial bond. Co–N (nitrogen atoms come from pyrazine moiety) bond distances are 2.190 Å and 2.183 Å. As per data available from the crystallographic database, the Co–N (pyridine) and average Co–N (pyrazine) bond distances are 2.075 Å and 2.1865 Å. From the data, it is evident that pyridine (presence of one nitrogen atom) can form a comparatively stronger bond compared to pyrazine (presence of two nitrogen atoms). Hence, Co–N (pyridine) bond distances are shorter than Co–N (pyrazine). The average metal-centric bond angles ∠N–Ni–N are 105.82° for complex **1** and 105.72° for complex **2**. In complex **1**, the two ligands (**N**_**3**_ **L^_1_^**) coordinate to the Fe­(III) center through six nitrogen donor atoms, generating four distinct five-membered chelate rings: [Fe1/N1/C5/C6/N2], [Fe1/N2/C10/C11/N3], [Fe1/N4/C25/C26/N5] and [Fe1/N5/C30/C31/N6]. These chelate rings exhibit bite angles of 80.59(10)° for ∠N1–Fe1–N2, 80.55(10)° for ∠N2–Fe1–N3, 80.51(10)° for ∠N4–Fe1–N5, and 80.69(10)° for ∠N5–Fe1–N6, indicating a nearly uniform coordination geometry (FeN_6_ chromophore) around the Fe­(III) ion. However, in complex **2**, the **N**_**3**_ **L^_1_^** ligand is able to bind with the Co­(II) ion to form two five-membered chelate rings, [Co1/N1/C4/C9/N5] and [Co1/N5/C13/C8/N3]. The corresponding bite angles, ∠N1–Co1–N5 = 75.89(9)° and ∠N3–Co1–N5 = 75.51(9)°, are noticeably smaller than those observed for complex **1**, reflecting the different coordination environment (CoN_3_O_2_Cl chromophore) and geometric preferences of the Co­(II) center. In complex **1**, the Fe­(III) ion is deviated by a distance of 0.023 Å toward the axial nitrogen atom (N34) from the basal plane (N15N7N9N25), whereas in complex **2**, the same is 0.014 Å toward the axial O1 atom from the basal plane (N35N30N12N19). The overall electrical charge of [Fe­(**HN**_**3**_ **L^_1_^**)­(**N**_**3**_ **L^_1_^**)] fragment is +4 (+3 charge of Fe­(III) and +1 charge from protonated N atom of imidazole unit of one **N**_**3**_ **L^_1_^** ligand), which is taken care by four noncoordinated chloride (Cl^–^) for complex **1**. In the case of complex **2**, the overall charge of [Co­(**HN**_**3**_ **L^_1_^**)­Cl­(H_2_O)_2_] is +2 (+2 charge of Co­(II) and +1 charge from the protonated N atom of imidazole unit of one **N_3_L^_1_^** ligand and −1 charge from Cl^–^), which is taken care by two noncoordinated Cl^–^. Here, in the present work, the title ligand (**N**_**3**_ **L^_1_^**) utilizes two pyrazine and one pyridine nitrogen in the coordination game. The solid-state structures of both the complexes are stabilized through the combination of π···π stacking and various types of hydrogen-bonding interactions (Tables S4 and S5).

The cationic part [Fe­(**N**_**3**_ **L** **^_1_^**)­(**HN**_**3**_ **L** **^_1_^**)]^4+^ of the asymmetric unit of complex **1** forms a dimeric assembly through a self-complementary face-to-face π···π stacking interaction between Cg(6) and Cg(12) of the adjacent units at (1-*x*,1-*y*,1-*z*) with an interplanar spacing of 3.9327(19) Å (Figure 3). These dimeric assemblies are further connected through the combination of three different H-bonding [C22–H22···Cl2 (165.40°) at (*x*,1/2-*y*,−1/2+*z*), C9–H9···Cl2 (167.80°) at (2-*x*,1-*y*,1-*z*), and N11–H11···Cl2 (173.90°) at (2-*x*,1-*y*,1-*z*)] interactions to produce a 2D supramolecular layered structure in *bc*-plane.

**Figure 3:**
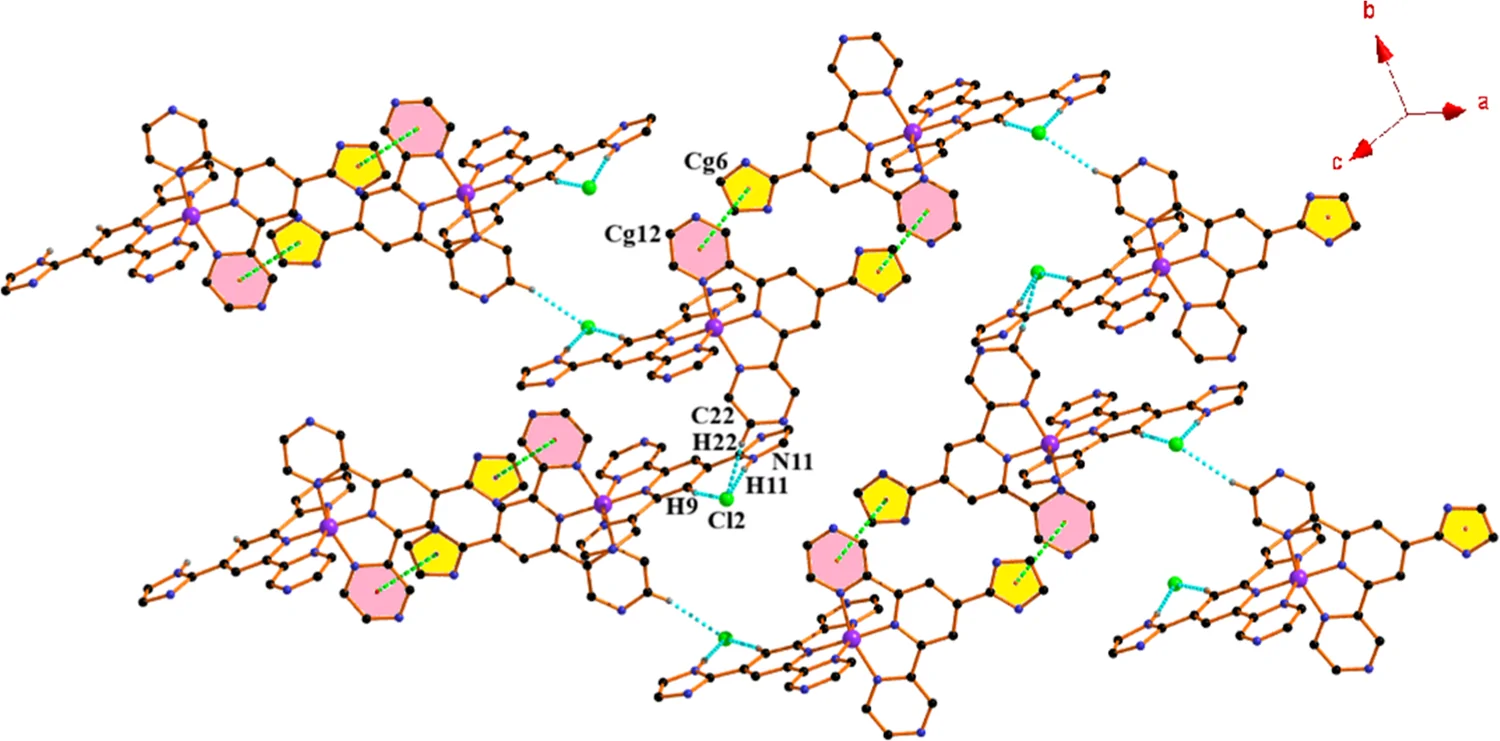
Formation of 2D-layered assembly through π···π stacking, C–H···Cl, and N–H···Cl hydrogen-bonding interactions in complex **1**.

The cationic part [Fe­(**N**_**3**_ **L** **^_1_^**)­(**HN**_**3**_ **L** **^_1_^**)]^4+^ of the asymmetric unit interacts with Cl2 in a zigzag fashion through three different H-bonding [C22–H22···Cl2(165.40°) at (*x*,1/2-*y*,−1/2+*z*), C9–H9···Cl2(167.80°) at (2-*x*,1-*y*,1-*z*), and N11–H11···Cl2(173.90°) at (2-*x*,1-*y*,1-*z*)] interactions. These interactions are repeated among themselves to extend a 1D polymeric supramolecular chain along the (010) direction, as depicted in Figure S4, Supporting Information. The cationic part [Fe­(**N**_**3**_ **L** **^_1_^**)­(**HN**_**3**_ **L** **^_1_^**)]^4+^ of the asymmetric unit of complex **1** connects further with Cl3 in a zigzag fashion through two different H-bonding [N14–H14A···Cl3 (161.50°), C2–H2···Cl3 (133.30°) at (*x*,1/2-*y*,−1/2+*z*)] interactions (Figure S5, Supporting Information). The repetition of these interactions results in the extension of a one-dimensional polymeric supramolecular chain along the (001) direction.

The cationic part [Co­(**HN**_**3**_ **L** **^_1_^**)­Cl­(H_2_O)_2_]^2+^ of the asymmetric unit of complex **2** forms a dimeric assembly through a self-complementary face-to-face π···π stacking interaction between Cg(3) and Cg(5) of the adjacent units at (1-*x*,1-*y*,1-*z*) with an interplanar spacing of 3.877(2) Å (Figure 4). These dimeric assemblies are further connected through the combination of five different H-bonding [O1–H1B···Cl3 (168°), C12–H12···Cl3 (169°) at (1-*x*,1-*y*,1-*z*), O4–H4B···Cl3(169°) at (1-*x*,-1/2+*y*,3/2-*z*), N7–H7A···Cl3 (177°) at (1-*x*,1-*y*,1-*z*), O4–HA···Cl1 (167°)] interactions to produce a 2D supramolecular layered structure in *ac*-plane.

**Figure 4:**
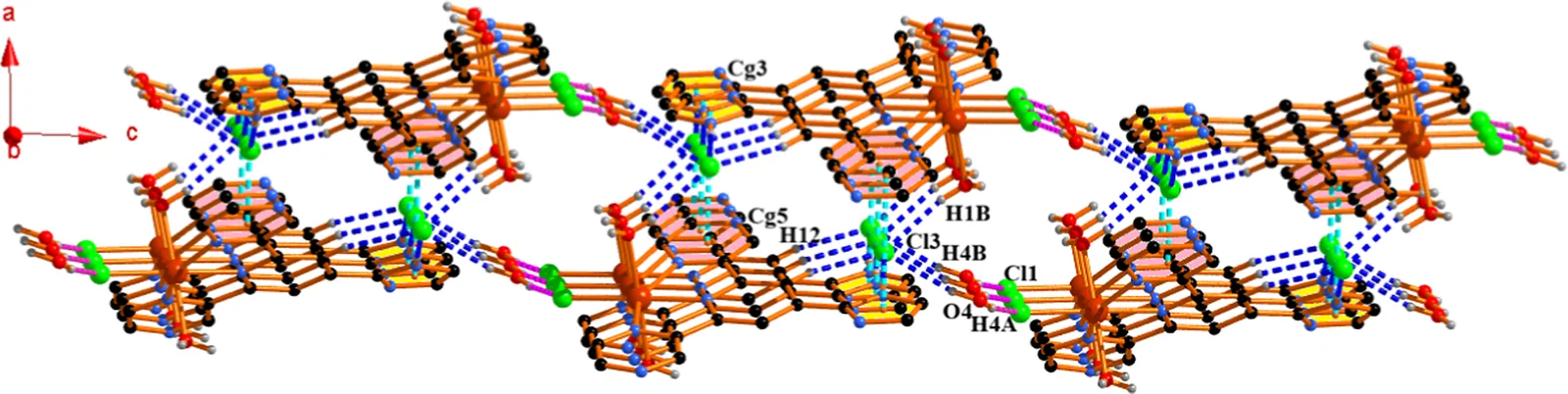
Two-dimensional (2D) supramolecular layer formation through π···π stacking, C–H···Cl, N–H···Cl, and O–H···Cl hydrogen-bonding in complex **2**.

Besides, the cationic part [Co­(**HN**_**3**_ **L** **^_1_^**)­Cl­(H_2_O)_2_]^2+^ of the asymmetric unit of complex **2** interacts with Cl2 through six different H-bonding [C10–H10···Cl2 (171°) at (*x*,1+*y*,*z*), N6–H6A···Cl2 (170°) at (*x*,1+*y*,*z*), O5–H5A···Cl2 (171°) at (−1+*x*,*y*,*z*), O5–H5B···Cl2 (164°) at (1-*x*,1-*y*,1-*z*), O2–H2B···O5 (168°) at (1 +*x*,*y*,*z*), and O2–H2A···N2 (171°) at (*x*,−1+*y*,*z*)] interactions (Figure S6, Supporting Information). These interactions are repeated among themselves to extend a 1D polymeric supramolecular tape along the (010) direction. In addition, the cationic part [Co­(**HN**_**3**_ **L** **^_1_^**)­Cl­(H_2_O)_2_]^2+^ of the asymmetric unit of complex **2** interacts with H_2_O molecules (both coordinated and noncoordinated) through six H-bonding O3–H3B···Cl1 (159°) at (1-*x*,1/2+*y*,3/2-*z*), O1–H1A···O3 (171°), O4–H4A···Cl1 (167°), O2–H2B···O5 (168°) at (1+*x*,*y*,*z*), O3–H3A···O4 (172°) at (*x*,1+*y*,*z*), and O3–H3B···O1 (126°) at (1-*x*,1/2 +*y*,3/2-*z*) interactions (Figure S7, Supporting Information). These interactions are repeated among themselves to extend a 1D supramolecular architecture along the (010) direction.

From a supramolecular point of view, the observed noncovalent interactions play a crucial role in directing the self-assembly of the coordination complexes into stable higher-dimensional architectures for both the complexes **1** and **2**. The cooperative combination of π···π stacking and hydrogen-bonding interactions generates well-organized 1D chains, supramolecular tapes, and 2D layered networks in the solid state. Chloride ions and lattice water molecules act as important supramolecular linkers and bridges, leading to the formation of robust hydrogen-bonded architecture. Overall, these weak interactions collectively govern crystal packing, stabilize the supramolecular assembly, and control the long-range organization of the crystal structure.

The crystal structures of the synthesized complexes **1** and **2** reveal that the coordinated ligand (**N**_**3**_ **L** **^_1_^**) acts as a tridentate chelating ligand, forming chelate rings around the metal centers. In both complexes, coordination of the heterocyclic ligand generates five-membered metallacyclic rings, which establish a well-defined coordination environment and significantly influence the overall geometry of the metal centers. Here, the tridentate ligand coordinates to the metal center twice in complex **1**, whereas it coordinates only once in complex **2**, while the remaining coordination sites of complex **2** are occupied by other coordinating ligands. Consequently, complex **1** contains four five-membered metallacyclic chelate rings formed by the tridentate ligand, while complex **2** possesses only two such chelate rings. This difference in the chelation pattern significantly influences the coordination environment around the metal centers, contributing to variations in their molecular geometry, stability, and supramolecular assembly. The formation of these chelate rings also enhances the thermodynamic stability of the complexes through the chelate effect (largely driven by the entropy). Moreover, the rigid coordination imposed by the tridentate ligand governs the spatial orientation of the coordinated molecules, thereby influencing the supramolecular assembly and the resulting intermolecular interactions within the crystal lattice. Thus, these rings reduce the likelihood of ligand dissociation and enhance the structural integrity of the complex.

### Hirshfeld Surface Analysis

To investigate the intermolecular interactions governing crystal packing, HS analysis^54–56^ and the corresponding two-dimensional (2D) fingerprint plots^57–59^ were generated using Crystal Explorer.^60^ Standard neutron-derived hydrogen-atom bond lengths were employed throughout the calculations. Each point on the HS is described by two geometrical parameters: the external distance (*d*_*e*_), corresponding to the distance from the surface to the nearest neighboring atom outside the molecule, and the internal distance (*d*_*i*_), representing the distance to the nearest atom within the molecule. These parameters are used to calculate the normalized contact distance (*d*_or_), which serves as an effective descriptor for identifying significant intermolecular interactions and is given by

$$d_{norm}=\frac{\left(d_{i}-{r_{i}}^{vdw}\right)}{{r_{i}}^{vdw}}+\frac{\left(d_{e}-{r_{e}}^{vdw}\right)}{{r_{e}}^{vdw}}$$

where *r*_*i*_^vdw^ and *r*_*e*_^vdw^ represent the van der Waals radii of the internal and external atoms, respectively. The value of *d*_norm_ reflects the nature of intermolecular contacts, with negative values indicating distances shorter than the sum of the van der Waals radii and positive values corresponding to longer separations. For visualization, the HS is mapped using a red–white–blue color scheme, where red identifies regions of close intermolecular contacts, white denotes contacts close to the van der Waals limit, and blue represents areas with comparatively longer intermolecular distances. Owing to its dependence on the crystallographic geometry, the HS generated from each CIF is unique for a given crystal structure.^61^

Hirshfeld surfaces of complexes **1** and **2** were generated and mapped with several surface properties, including *d*_norm_, *d*_i_, *d*_e_, shape index, curvedness, and fragment patches (Figures 5 and 6). Each of these surface mappings provides complementary information regarding the intermolecular interactions governing the crystal packing. The shape index surface is particularly useful for identifying donor–acceptor contacts and recognizing complementary surface features associated with specific intermolecular interactions, whereas the curvedness surface illustrates the degree of local surface curvature, thereby partitioning the molecular surface into distinct regions. Fragment-patch mapping highlights the spatial relationship between neighboring molecules by assigning different color patches according to their proximity to the reference molecule. To facilitate visualization of the molecular framework enclosed by the HS, all surfaces were rendered with partial transparency while maintaining a common molecular orientation for both structures. The overall pattern of intermolecular contacts was further examined using two-dimensional (2D) fingerprint plots, which combine the internal (*d*_i_) and external (*d*_e_) distance parameters to provide a quantitative representation of all close contacts present within the crystal (Figures 5 and 6). Characteristic intermolecular interactions appear as well-defined features or spikes in these plots, allowing their nature and relative significance to be readily distinguished. In addition, the fingerprint plots were decomposed into individual atom-pair contributions, enabling the identification and quantitative assessment of specific interaction types that overlap in the complete fingerprint map. The HS analysis, together with the decomposed fingerprint plots, confirms the occurrence of the intermolecular interactions discussed above and demonstrates their collective role in stabilizing the crystal packing and promoting the three-dimensional supramolecular architecture of the complexes.

**Figure 5:**
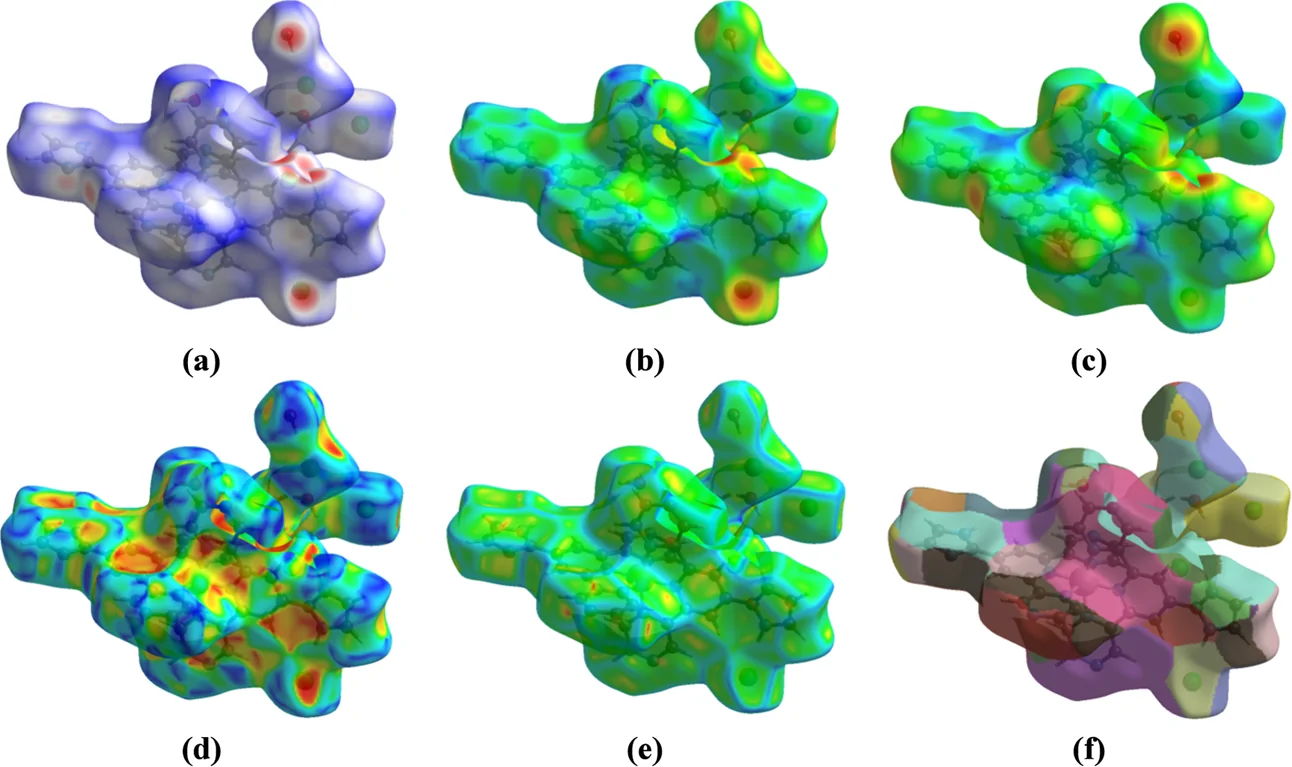
Hirshfeld surfaces mapped with (a) *d*_norm_, (b) *d*_*e*_, (c) *d*_*i*_, (d) shape-index, (e) curvedness, and (f) fragment patch for complex **1**.

**Figure 6:**
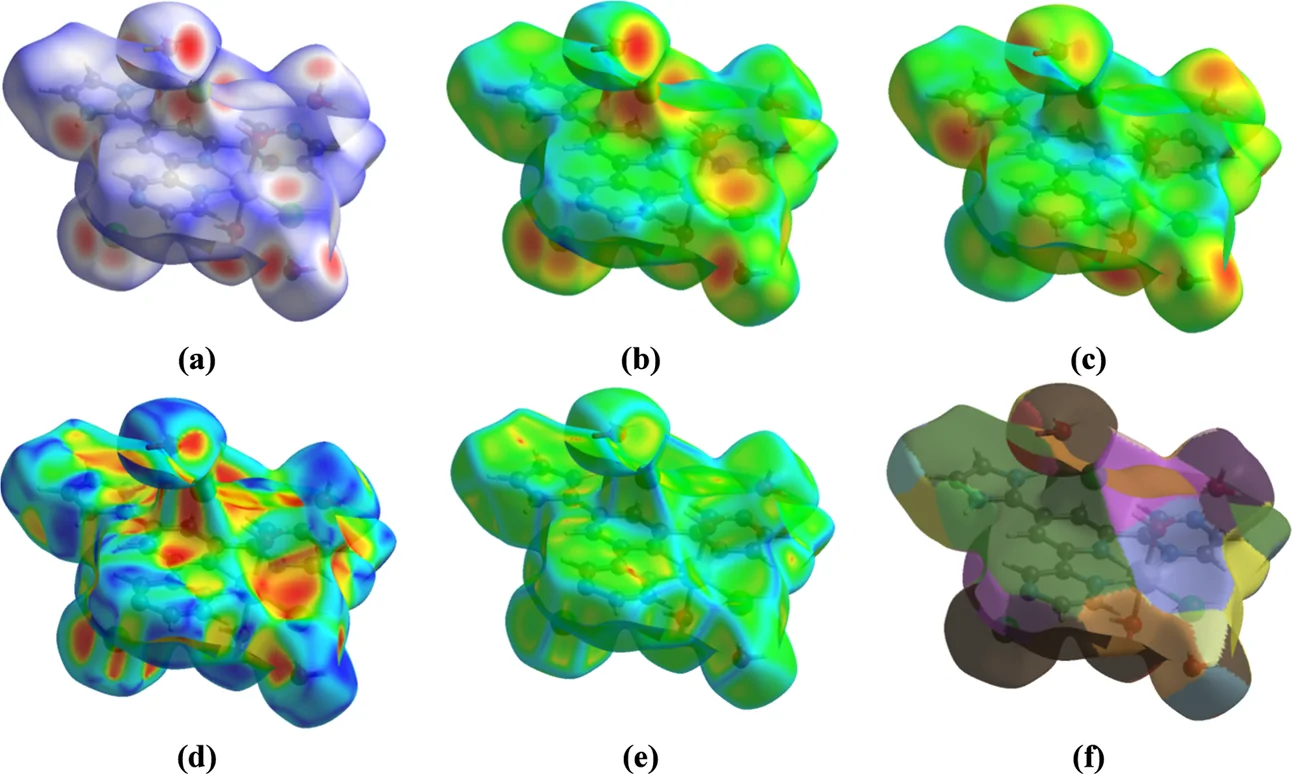
Hirshfeld surfaces mapped with (a) *d*_norm_, (b) *d*_*e*_, (c) *d*_*i*_, (d) shape-index, (e) curvedness, and (f) fragment patch for complex **2**.

The decomposed two-dimensional fingerprint plots provide a quantitative comparison of the intermolecular contacts responsible for the crystal packing of complexes **1** and **2** (Figures 7 and 8). In both structures, H···H contacts constitute the largest contribution to the HS, accounting for 31.0% in complex **1** and 32.2% in complex **2**, to the overall crystal packing of the two complexes.

**Figure 7:**
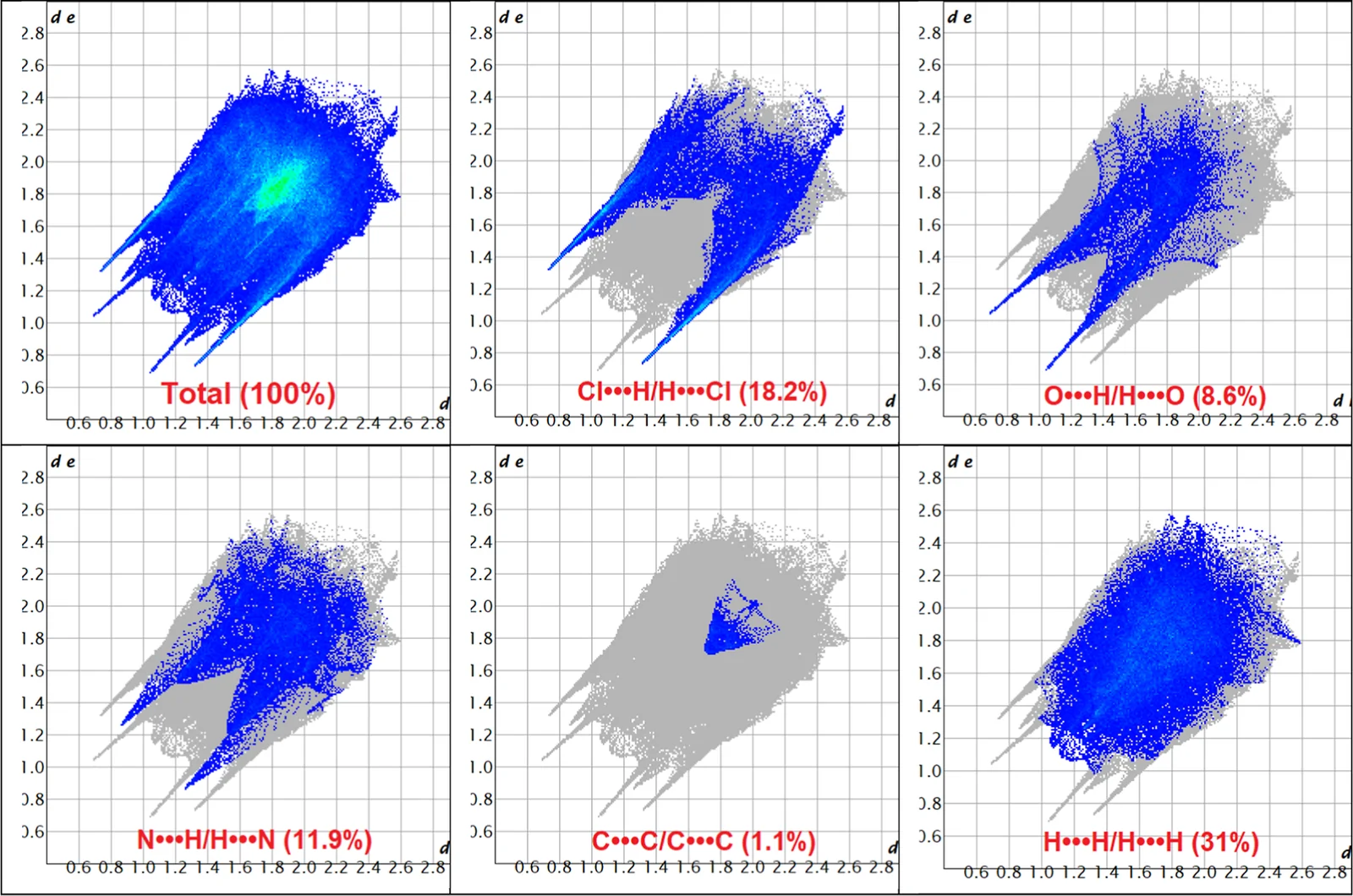
Fingerprint plot (full) and decomposed plots for each individual interaction in complex **1**.

**Figure 8:**
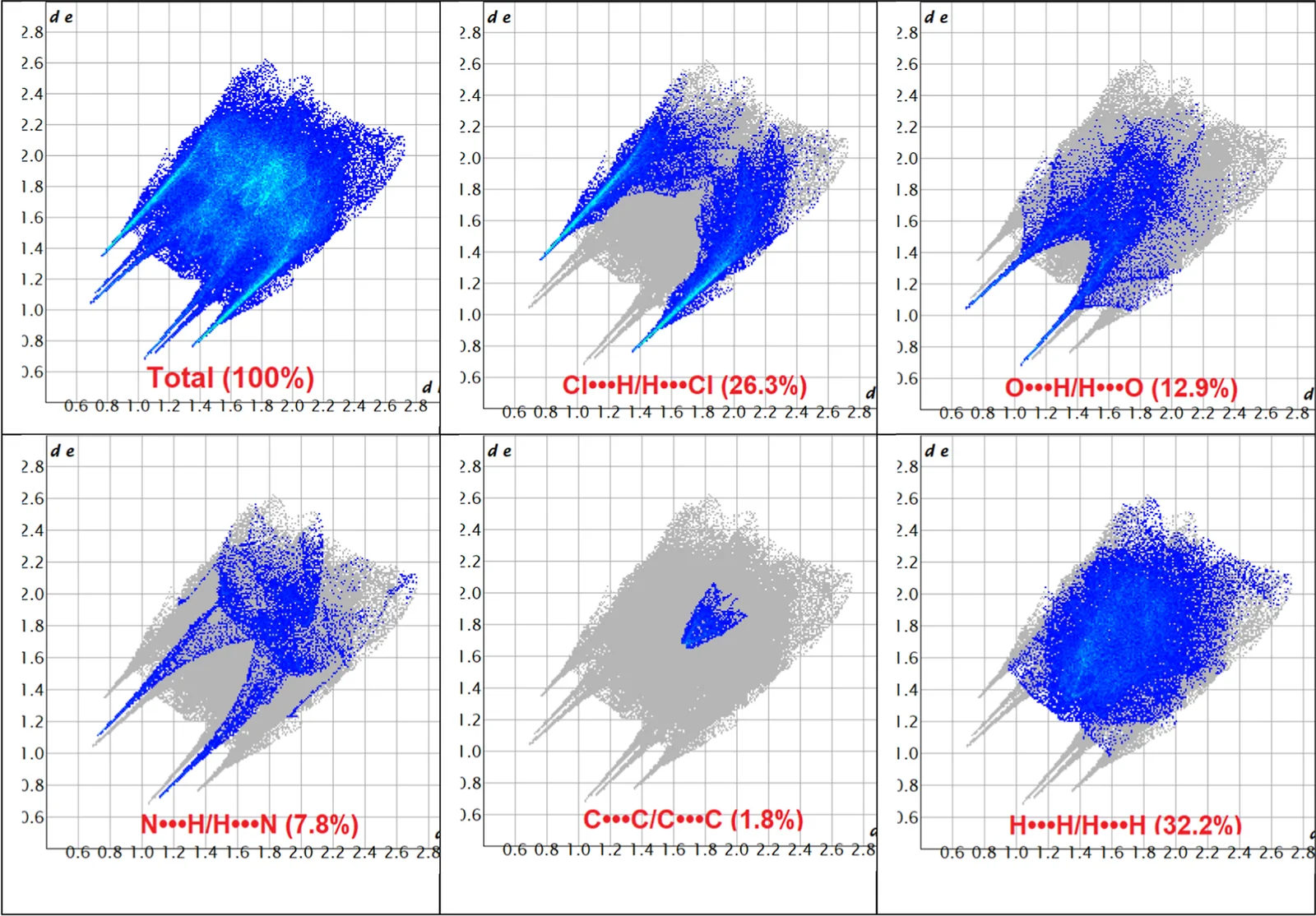
Fingerprint plot (full) and decomposed plots for each individual interaction in complex **2**.

Among the heteroatom-involving interactions, Cl···H/H···Cl contacts represent the second most significant contribution in both complexes. These interactions appear as two well-defined spikes in the fingerprint plots and contribute 18.2% to the HS of complex **1**, whereas their contribution increases markedly to 26.3% in complex **2**. This notable enhancement suggests that chloride-mediated hydrogen-bonding interactions play a more dominant role in stabilizing the crystal lattice of complex **2**. The O···H/H···O contacts are also characterized by a pair of sharp spikes in the fingerprint plots, reflecting the presence of strong hydrogen-bonding interactions involving oxygen atoms. These contacts account for 8.6% of the HS in complex **1** but increase to 12.9% in complex **2**, demonstrating a greater participation of oxygen-containing hydrogen bonds in the supramolecular assembly of the latter. In contrast, the N···H/H···N interactions contribute more significantly to the crystal packing of complex **1** (11.9%) than complex **2** (7.8%). The reduced contribution in complex **2** indicates that nitrogen-centered hydrogen-bonding interactions are comparatively less important, with the packing instead being dominated by Cl···H/H···Cl and O···H/H···O contacts. The C···C contacts, which are commonly associated with aromatic π···π stacking interactions, make only a minor contribution in both structures. Nevertheless, the contribution is slightly higher for complex **2** (1.8%) than for complex **1** (1.1%), implying a somewhat greater extent of π···π interactions in the former.

Overall, the fingerprint plot analysis reveals that complex **2** exhibits substantially stronger Cl···H/H···Cl and O···H/H···O interactions, together with a modest increase in C···C contacts, whereas complex **1** is characterized by a relatively larger contribution from N···H/H···N interactions. These differences indicate that, despite sharing similar types of intermolecular interactions, the relative importance of individual contact types varies between the two complexes, resulting in distinct supramolecular packing environments and stabilization mechanisms.

### Theoretical Analysis for Noncovalent Interactions

In this section, we provide a detailed theoretical investigation of the noncovalent interactions that stabilize the solid-state structure of complex **2**. We have employed MEP surface analysis on a neutral fragment of complex **2** to identify the nucleophilic and electrophilic regions of the molecule. To gain deeper insights into the nature and strength of the supramolecular contacts, QTAIM was utilized to analyze the hydrogen-bonding (H-bonding) networks within several representative assemblies of complex **2** with the lattice water molecules. Furthermore, we have examined the occurrence of anion···π and lone-pair···π interactions involving the chloride anions and water molecules, respectively. The latter interactions were further characterized using Noncovalent interaction (NCI plot) analysis to visualize the stabilizing regions between the interacting moieties.

The theoretical study focuses exclusively on complex **2** because of several practical and computational considerations. Primarily, complex **2** presents a more compact system, where the impact of extensive H-bonding and auxiliary interactions can be more clearly isolated and evaluated. In contrast, complex **1** poses significant computational challenges; its larger molecular size and the requirement to model assemblies involving multiple units would result in prohibitively high computational costs. Additionally, the presence of Fe­(III) centers in complex **1** introduces a high number of unpaired electrons, which significantly complicates the electronic convergence and reliability of calculations at the PBE0-D4/def2-TZVP level of theory employed in this work. Therefore, complex **2** serves as a more accessible and representative model for understanding the supramolecular forces at play in these types of imidazole-pyrazinyl pyridine systems.

The MEP surface of complex **2** was analyzed using a neutral system where two chloride anions are located in the molecular plane and hydrogen-bonded to the imidazolium NH groups (see Figure 9). The calculated MEP reveals that the global minima are located at the noncoordinated Cl atoms with values of −55.8 kcal/mol, followed by the nitrogen atoms of the pyrazine rings at −23.1 kcal/mol and the coordinated Cl atom at −22.5 kcal/mol. The MEP maximum is located at the H atoms of the coordinated water molecules at 82.8 kcal/mol, which point toward the central pyridine ring. Other H atoms on these water molecules show a value of 74.4 kcal/mol. These large positive values align with the network of hydrogen bonds involving these water molecules. Finally, the π–surface of the organic ligand is positive throughout, ranging from 30.1 kcal/mol over the imidazolium ring to 38.9 kcal/mol over the pyridine. The value over the pyrazine rings is 34.5 kcal/mol. This consistent positive potential across the surface of the ligand explains its ability to participate in anion···π and lone-pair···π interactions.

**Figure 9:**
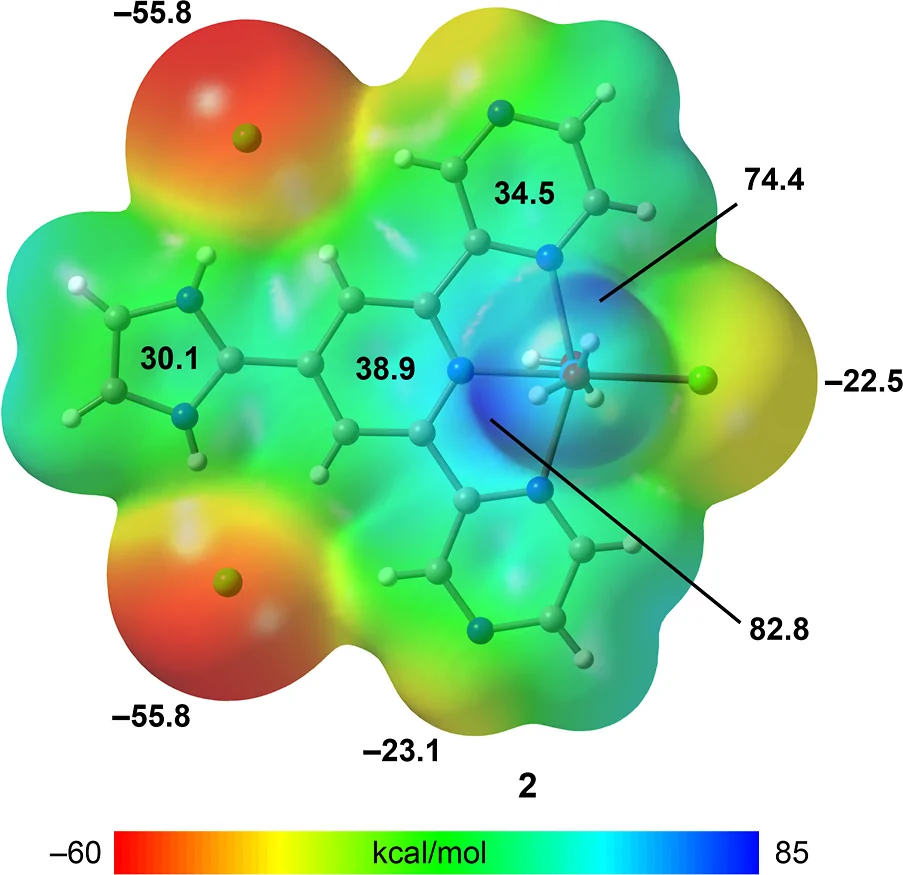
MEP surface of a neutral fragment of complex **2** calculated at the PBE0-D4/def2-TZVP level of theory. The MEP values at selected points (minima and maxima) and over the aromatic rings are indicated in kcal/mol. The two noncoordinated chloride anions are included to maintain the neutrality of the system.

The supramolecular architecture of complex **2** was further investigated using the QTAIM method to characterize and quantify the hydrogen-bonding networks. This approach allows for the identification of specific H bonds through the presence of bond critical points (BCPs) and bond paths, while also providing a means to evaluate the strength of individual contacts independently of the overarching Coulombic attractions inherent to the ion-pair nature of the assemblies.

Figure 10a illustrates a tetrameric *R*_4_^4^(8) synthon composed of two chloride anions and two lattice water molecules. This [(H_2_O)_2_Cl_2_]^2–^ cluster acts as a supramolecular bridge, connecting two molecules of complex **2** through the oxygen atoms of the cluster. The formation energy of this *R*_4_^4^(8) synthon is calculated at −13.0 kcal/mol, underlining its structural significance in the solid-state arrangement. The individual H bonds within the synthon are characterized by BCPs (represented by red spheres) and associated bond paths. Notably, the H bonds linking the coordinated water molecules of the metal complex to the synthon represent the strongest contacts in this assembly, with an energy of −7.3 kcal/mol.

**Figure 10:**
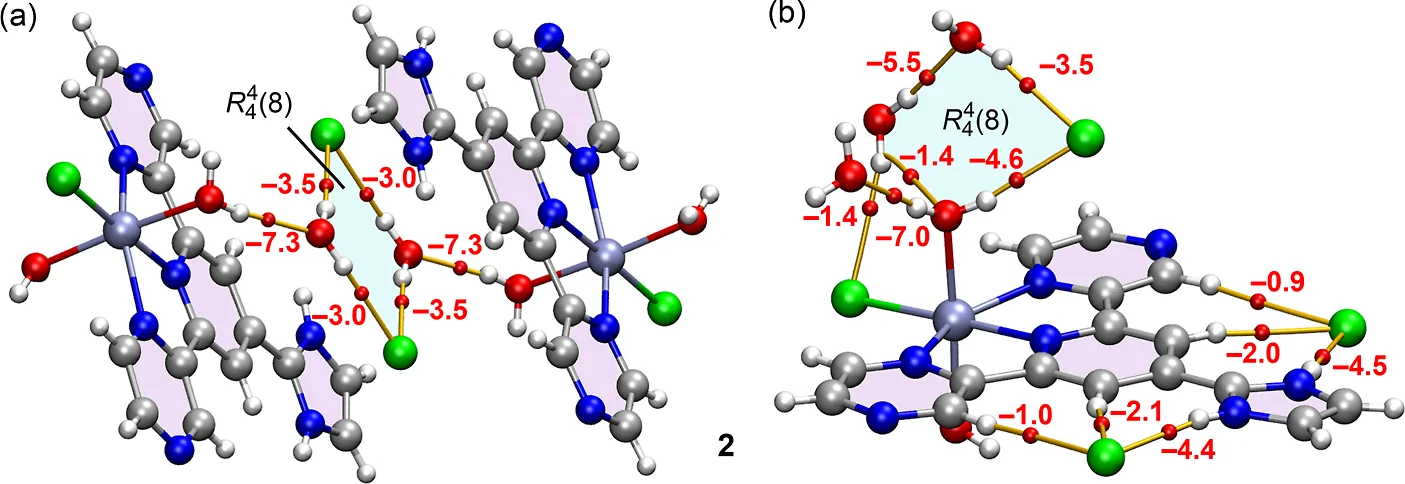
QTAIM analysis of the H-bonding assemblies in complex **2** calculated at the PBE0-D4/def2-TZVP level of theory. (a) Supramolecular cluster involving the formation of a *R*_4_^4^(8) synthon between lattice water molecules and chloride anions. (b) Detailed H-bonding network involving the coordinated water molecule and trifurcated H bonds between the coplanar chloride anions and the organic ligand. BCPs are represented by red spheres and bond paths by orange lines. Individual H-bond energies and total synthon formation energies are indicated in kcal/mol.

The QTAIM analysis presented in Figure 10b focuses on the H-bonding network involving the coordinated water molecule positioned opposite to the cluster described above. This coordinated water molecule functions as a donor in two strong H bonds and as an acceptor in one weaker interaction. In this region, another *R*_4_^4^(8) synthon is identified, involving two lattice water molecules, one chloride anion, and the coordinated water molecule. The formation energy for this assembly is −15.0 kcal/mol, indicating it is energetically more favorable than the cluster shown in Figure 10a.

Additionally, the interactions of the chloride anions that are coplanar with the ligand were examined. QTAIM analysis reveals that each chloride ion participates in a trifurcated H-bonding arrangement. In addition to the primary N–H···Cl hydrogen bonds, which exhibit strengths of −4.4 and −4.5 kcal/mol, the chloride ions form two additional, weaker C–H···Cl H-bonds with the hydrogen atoms of the adjacent pyridine and pyrazine rings. These auxiliary contacts exhibit strengths ranging from −0.9 to −2.1 kcal/mol, further contributing to the stabilization of the supramolecular network.

To further demonstrate the ability of the ligand in complex **2** to participate in π-interactions with electron-rich moieties, we have combined QTAIM analysis with the NCI plot method, as illustrated in Figure S8, Supporting Information. While QTAIM provides quantification, the NCI plot is particularly well-suited for visualizing the extended nature of π-interactions in real space, offering complementary information regarding the spatial extent and relative strength of the contact. The crystal structure shows that a chloride anion and a lattice water molecule are positioned directly above and below the central pyridine ring, respectively. These interactions occur in concert with strong H bonds, the quantification of which is established in Figure 10. Both the anion···π interaction involving the chloride and the lone-pair···π (lp···π) interaction involving the water molecule are distinctly characterized by a BCP and a bond path connecting the Cl or O atoms to one of the carbon atoms of the pyridine ring.

The extended visual nature of these contacts is more clearly delineated by the reduced density gradient (RDG) isosurfaces generated by the NCI plot analysis. For the anion···π interaction, a significant green RDG surface is observed, which partially embraces the adjacent five-membered Co-chelate ring, highlighting the extensive stabilizing region. In contrast, the RDG isosurface corresponding to the lone-pair···π interaction is significantly smaller and more localized, suggesting a comparatively less-influential interaction.

To evaluate the strength of this multi-interaction assembly, we used a neutral fragment, defined as [Co­(**HN**_**3**_ **L**_**1**_)­(H_2_O)_2_Cl]­Cl_2_, to minimize the overestimation of binding energy that would arise from pure coulombic attraction if fully charged species were used as monomers. Despite this, the formation energy for this assembly is very large at −59.2 kcal/mol. This high value reflects the robust overall stabilization provided by the strong electrostatic attraction between the anionic Cl^–^ species and the protonated ligand, further augmented by the coordination of the Co­(II) cation, which significantly increases the π-acidity of the aromatic ligand framework.

### TD-DFT Study

To gain deeper insights into the electronic excitations governing the optical absorption and establish a direct structure–property relationship, TD-DFT calculations were performed on both complexes.^62^ The theoretical UV–Vis spectra were computed at the CAM-B3LYP-D4/def2-TZVP level and employing an implicit solvent model (see Computational Methods in Supporting Information) to mimic experimental conditions. Natural transition orbitals (NTOs) were visualized to characterize the electron density redistributions during electronic excitations. NTOs provide a minimal and intuitive representation of electronic excitations by identifying the most significant occupied-to-virtual orbital transitions, thereby simplifying visualization and interpretation compared to examining all individual molecular orbitals.^63^ Detailed TD-DFT results for the lowest-energy and most intense transitions are presented in Table 1 and visualized in Figure 11.

**Table 1:** TD-DFT-Calculated Excitation Energies, Oscillator Strengths, and Orbital Composition of the Electronic Transitions for Complexes **1** and **2** ^a^

excitation	composition (%)	spin	λ (nm)	*f*_osc_	type
**1**					
S_0_ → S_1_	HOMO → LUMO +2 (71.0%)	β	512.8	0.1375	LLCT
S_0_ → S_37_	HOMO–4 → LUMO (28.8%)	α	305.2	0.7540	LMCT &
	HOMO–7 → LUMO (13.6%)	β			LLCT
	HOMO–3 → LUMO (25.4%)	β			
**2**					
S_0_ → S_5_	HOMO → LUMO +6 (33.5%)	β	595.8	0.0010	MMCT
	HOMO → LUMO +7 (21.9%)	β			
S_0_ → S_17_	HOMO–1 → LUMO (10.6%)	α	301.8	0.1892	LLCT &
	HOMO–2→ LUMO (34.3%)	β			MLCT

a Only orbital contributions accounting for ≥ 10% of the total excitation are listed; NTOs in Figure 11 represent all α- and β-spin contributions.

**Figure 11:**
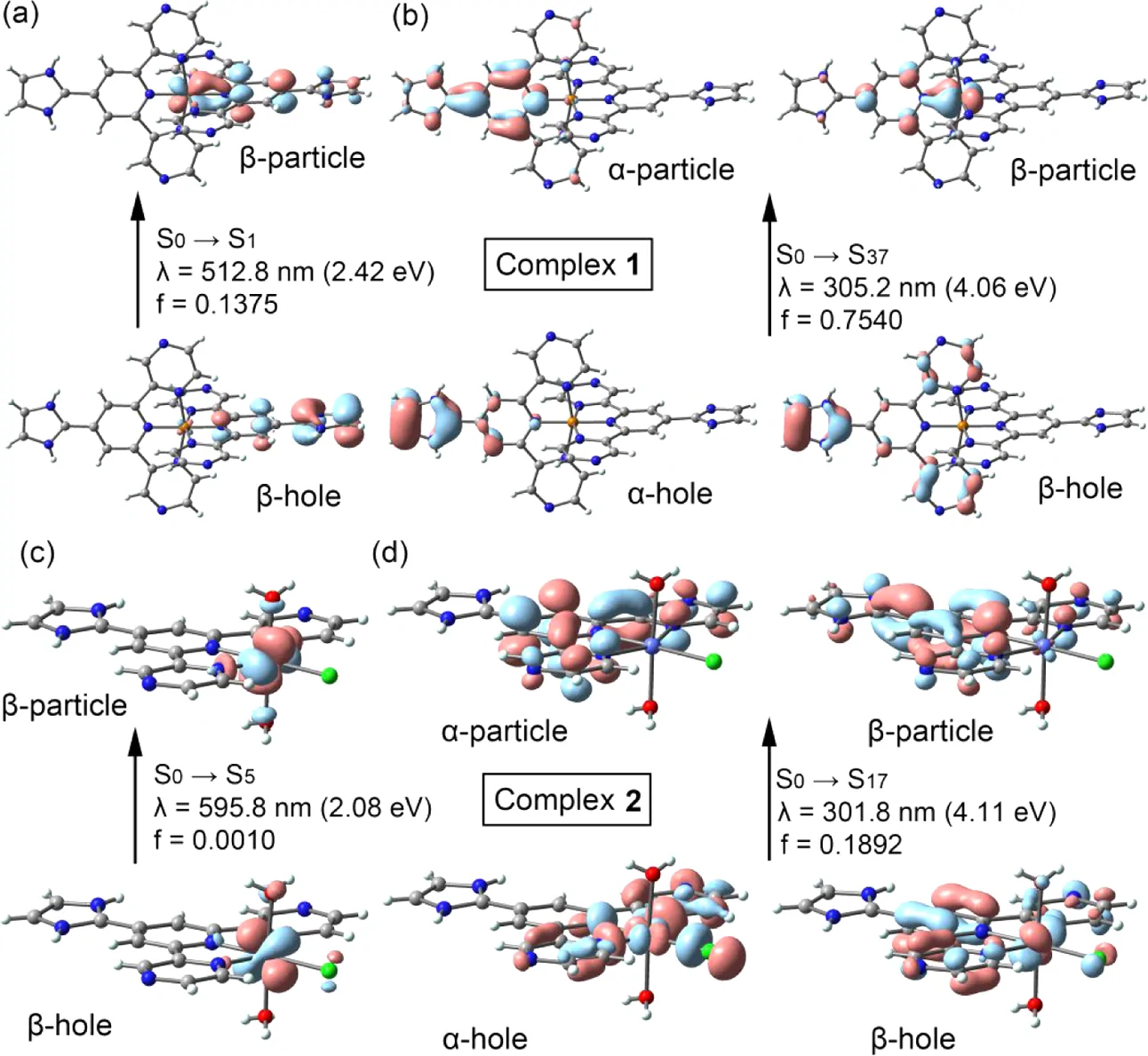
NTO analysis of electronic excitations in complexes **1** (a,b) and **2** (c,d). Panels show particle (acceptor orbital) and hole (donor orbital) for each transition and separate for α- and β-transitions. Greek symbols α and β denote α- and β-spin contributions.

In complex **1**, the lowest-energy electronic transition (S_0_ → S_1_) appears at 512.8 nm (2.42 eV) with a modest oscillator strength (*f* = 0.1375). This transition is primarily composed of a ligand-to-ligand charge-transfer (LLCT) character, with the dominant contribution being HOMO → LUMO +2 (71.0%) transition. A secondary component from the iron center (HOMO–4 → LUMO, 28.8%) indicates a small ligand-to-metal charge-transfer (LMCT) contribution, confirming the participation of Fe d-orbitals in the lowest electronic excitation. The calculated band gap of 2.42 eV is in good agreement with the experimentally derived optical band gap of 2.02 eV, with the slight deviation attributable to the well-known tendency of TD-DFT to overestimate excitation energies. The relatively low oscillator strength characterizes this transition as a weakly allowed transition, consistent with the weak absorption tail observed in the experimental UV–Vis spectrum at this wavelength.

In complex **2**, the lowest-energy excitation (S_0_ → S_5_) occurs at 595.8 nm (2.08 eV) with a low oscillator strength (*f* = 0.0010), indicating a very weak absorption at this wavelength. This transition is primarily composed of metal-to-ligand metal charge transfer (MMCT), arising predominantly from HOMO → LUMO +6 (beta, 33.5%) and HOMO → LUMO +7 (beta, 21.9%) transitions. The calculated energy of 2.08 eV is remarkably close to the experimental band gap of 2.16 eV, indicating excellent agreement between theory and experiment for complex **2**.

The most intense absorption bands centered near 300 nm are associated with high-energy electronic transitions and dominate the optical absorption profile. These transitions are characterized by higher oscillator strengths and primarily involve π → π* transitions within the aromatic ligand framework, with varying degrees of metal involvement (see Figure 11).

In complex **1**, the most intense transition (S_0_ → S_37_) appears at 305.2 nm (4.06 eV) with a substantial oscillator strength (*f* = 0.7540), reflecting strong absorption in this spectral region. This state arises from a complex superposition of LLCT transitions, predominantly involving HOMO–3 → LUMO (25.4%) (beta), HOMO–4 → LUMO (alpha, 28.8%), and HOMO–7 → LUMO (beta, 13.6%) excitations. The NTO visualization (Figure 11b) reveals that the electron density redistribution occurs primarily within the aromatic ligand framework, involving transitions from the occupied π-orbitals of the imidazole moiety to the ligand-based π*-orbitals mostly localized in the adjacent pyridine ring, with a small contribution of the metal in the β-NTOs. This assignment confirms that the intense absorption at ∼305 nm arises mainly from π → π* transitions localized on the conjugated heterocyclic ligand. In complex **2**, the most intense transition (S_0_ → S_17_) is located at 301.8 nm (4.11 eV) with an oscillator strength (*f* = 0.1892) that is lower than that in complex **1**. This excitation is predominantly composed of LLCT transitions, with the primary contribution from HOMO–2 → LUMO (beta, 34.3%) and a secondary contribution from HOMO–1 → LUMO (alpha, 10.6%). The NTO analysis (Figure 11c) demonstrates that electron density is initially localized on the ligand π-framework (particularly the coordinated pyridine and pyrazine rings) and is transferred to unoccupied π*-orbitals of the conjugated system upon excitation, with the participation of the metal center in the hole orbitals, thus suggesting some MLCT character of this transition. The lower oscillator strength compared to complex **1** suggests that the absorption intensity is reduced, which correlates with the experimental observation that complex **2** exhibits weaker absorption in this spectral region compared to complex **1**. The difference in f-values between the two complexes likely reflects the different coordination environments: the octahedral Fe­(III) complex with two coordinated ligands (complex **1**) provides a more symmetric and extended conjugation pathway compared to the more distorted coordination geometry in complex **2**, resulting in more efficient electronic coupling and stronger oscillator strength for the π → π* transitions.

### Optical Study

Optical band gaps of the concerned complexes were derived from the UV–Vis absorption spectra. Figure 12a shows Tauc’s plot [(αhν)^2^ vs hν] with respect to complex **1** (blue curve) and complex **2** (red curve), while the inset figure shows the corresponding UV–Vis absorption spectra. Following the Tauc relation as given by eq 1, we have calculated the optical band gap of the complexes

1 $$\left(\alpha h\nu \right)=A{\left(h\nu -E_{g}\right)}^{n}$$

**Figure 12:**
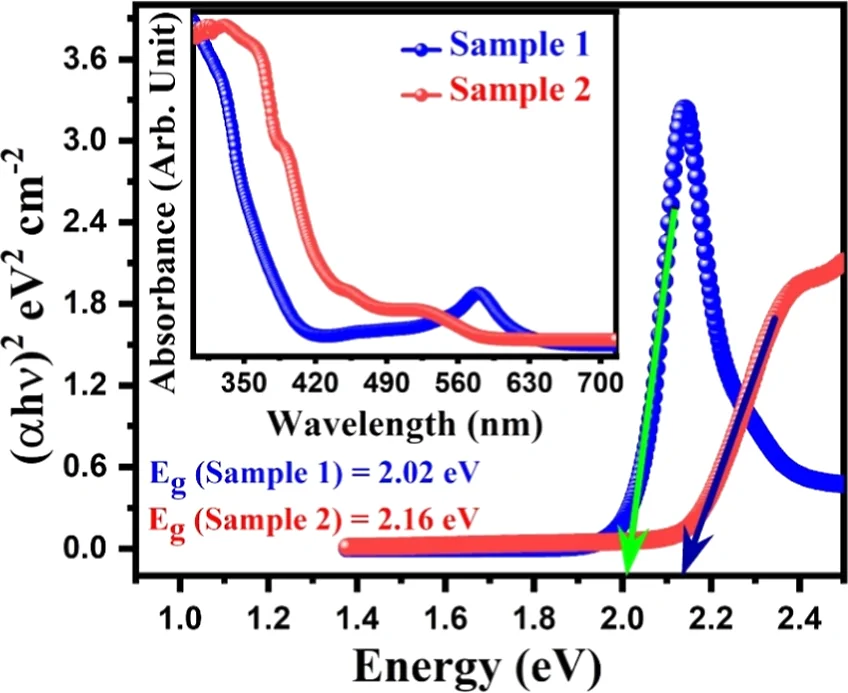
Tauc plots [(α*h*ν)^2^ vs *h*ν] from complex **1** (blue color) and complex **2** (red color) to determine the optical band gaps. The inset figure represents the corresponding UV–V is absorption spectra from these complexes.

where α is the absorption coefficient, *h*ν is the photon energy, *A* is a proportionality constant (wavelength-independent), *E*_*g*_ stands for the optical band gap of the complex, and we have taken the coefficient *n* = 1/2 considering direct band-to-band transition. From the Tauc’s plot [(αhν)^2^ vs hν], the optical band gaps (*E*_*g*_) of the complexes **1** and **2** are determined to be 2.02 and 2.16 eV, respectively. These results highlight the potential of the complexes for efficient optoelectronic device applications in the visible wavelength regime, provided they offer reasonably good charge transport properties.

### Electrical Study

For both the devices, electrical characterizations (current (*I*) vs voltage (*V*) characteristics) were done at room temperature, in the dark conditions. Figure 13a shows the schematic representation of the sandwiched Schottky device architecture and the electrical measurement unit. Figure 13b,c represents the *I*–*V* characteristics (both in linear scale and semilogarithmic scale) exhibited by device **1** and device **2,** respectively, demonstrating the rectifying feature of Schottky diodes. It is found that at ±1 V, device **1** (made of complex **1**) offers a rectification ratio (ratio of forward current to reverse current) of ≈66, which is nearly four times higher than that of device **2** (≈17). Device **2** exhibits even a lower rectification ratio of ≈14 at ±5 V. So, it can be concluded that device **1** will be efficient for rectifier-based device applications in comparison to device **2**. Analyzing the linear portion of the *I* vs *V* graphs (shown in Figure 13b,c), room-temperature conductivities (σ) were found to be 9.2 × 10^–5^ S m^–1^ and 1.14 × 10^–5^ S m^–1^, respectively, for complexes 1 and 2. Although device **1** (i.e., complex **1**) outperforms device **2** (i.e., complex **2**) in terms of rectification ratio and charge transport properties, still its performance falls short to meet the fundamental requirements of effective optoelectronic device applications.

**Figure 13:**
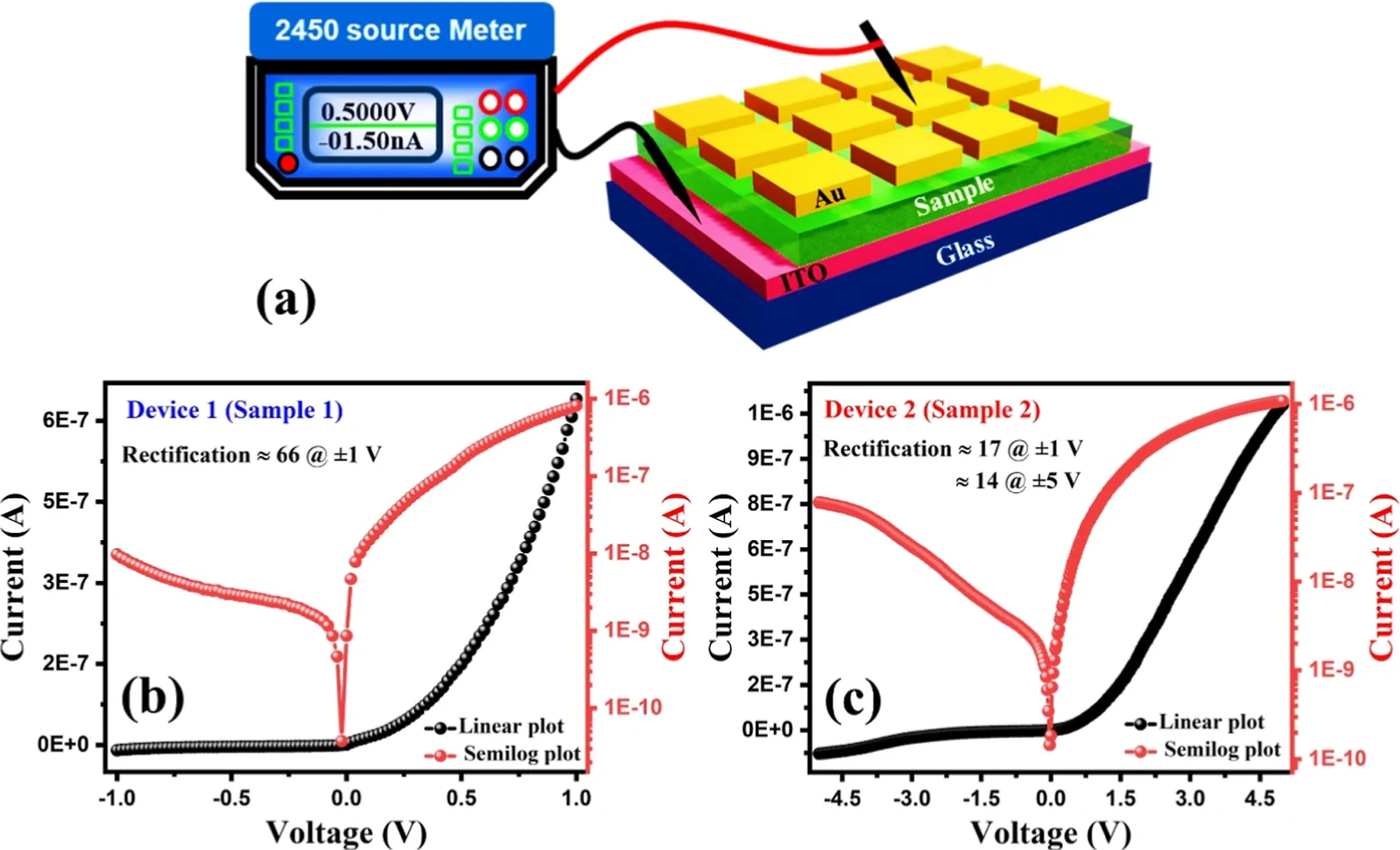
(a) Schematic representation of the Schottky device (diode) architecture. (b,c) Current–voltage characteristic curves (linear scale and semilog scale) obtained from device **1** and device **2**, respectively, under dark conditions.

We have further analyzed these results to gain comprehensive understanding about these rectifying *I*–*V* characteristics and charge transport mechanisms. These nonlinear, rectifying *I*–*V* characteristics can be elucidated by employing the Richardson–Schottky equation which incorporates thermionic emission theory and diffusion of carriers over barriers.^64,65^ As the concerned complexes are wide- band-gap in nature, to interpret the obtained *I*–*V* characteristics, we can apply thermionic emission theory-based Schottky diode eq 2:^65,66^

2 $$I=I_{s}\left[\exp\left(\frac{qV}{\eta kT}\right)-1\right]$$

where *I* is forward current, *I*_*s*_ is reverse saturation current, *V* is the applied voltage,η is the ideality factor, *q* is the elementary charge, *k* is the Boltzmann constant, and *T* is the absolute temperature. However, reverse saturation current (*I*_*s*_) can be written as^64–66^

Considering the diode series resistance (*R*_*s*_) in the account, eq 2 in the forward voltage regime (for $>\frac{3k_{B}T}{q_{e}}$ ) can be written as^64,67^

3 $$I=I_{s}\left[\exp\left(\frac{q_{e}\left(V-IR_{s}\right)}{\eta k_{B}T}\right)\right]$$

where *IR*_*s*_ signifies voltage drop across the series resistance *R*_*s*_. We have determined Schottky junction properties like ideality factor (η) and the series resistance (*R*_*s*_) of both the devices using eq 4 that is being derived by Cheung et al. combining the above two equations.^38,67^

4 $$G\left(I\right)=\frac{dV}{d\left(\ln I\right)}=\frac{\eta k_{B}T}{q_{e}}+IR_{s}$$

Now, from the slope and *Y*-axis intercept of linear *G*(*I*) vs *I* plots shown in Figure 14a,b, the values of series resistance (*R*_*s*_) and the ideality factor (η) are obtained, corresponding to device **1** and device **2,** respectively. The ideality factors (η) for device **1** and device **2** are found to be 4.1 and 4.9, respectively. It is evident that the obtained η values for both devices are substantially deviated from the ideal case (η = 1), indicating the formation of non-ideal metal–semiconductor junctions in these Schottky devices, presumably due to the presence of defects or trap states at the interfaces.^38,64^ Structural disorder in the complexes adversely influences the thin-film quality by inducing increased surface roughness and irregularities, which in turn foster the emergence of more defect states at the metal–semiconductor junctions. All these aspects can be corroborated to the significant deviation of η values from the ideal situation.^38,64^ Nevertheless, other factors like barrier tunneling and surface contamination can also play crucial role in the nonideal behavior of the fabricated Schottky diodes.^38,68^ At the same time, the series resistance (*R*_*s*_) values corresponding to the devices **1**and **2** are found to be 1.2 × 10^3^ (±110) kΩ and 10.2 × 10^3^ (±150) kΩ, respectively (see Figure 14a,b). Hence, device **1** (complex **1**) demonstrates almost nine times lower resistance than device **2** (complex **2**), signifying the formation of a relatively better metal–semiconductor junction in device **1**. As a result, comparatively efficient charge transportation is expected from device 1.^38,69^ Consequently, nearly eightfold higher conductivity offered by device **1** is further validated by its lower series resistance compared to device **2**.

**Figure 14:**
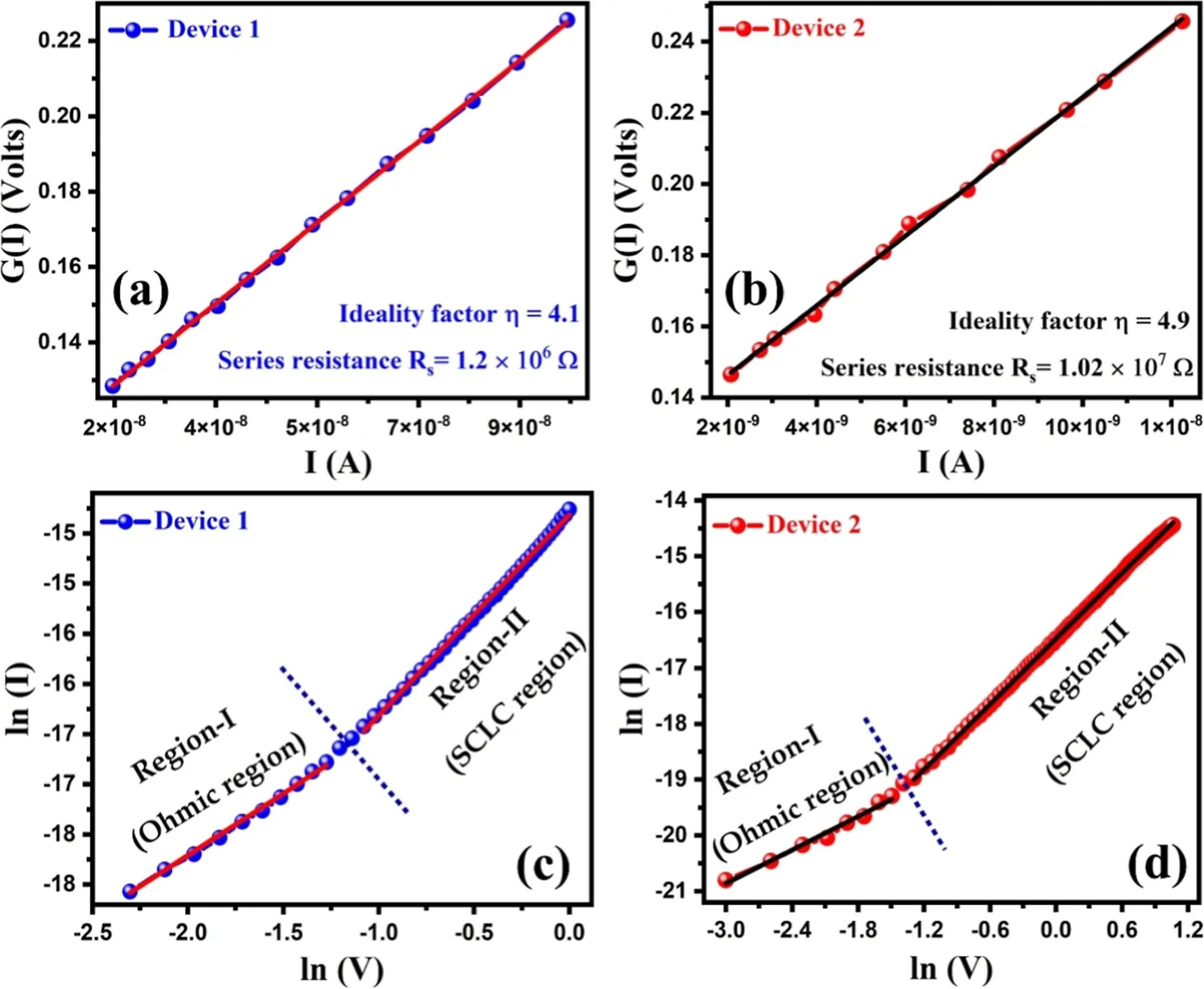
(a,b) G (I) vs. *I* characteristics under dark conditions corresponding to device **1** (complex **1**) and device **2** (complex **2**), respectively. (c,d) Forward-bias ln­(*I*) vs ln­(*V*) graphs of device **1** and device **2**, respectively.

For comprehensive understanding on the charge transportation mechanisms across the metal–semiconductor interface, forward-bias ln­(*I*) vs ln­(*V*) curves have been plotted for both the devices, as shown in Figure 14c,d, respectively. The presence of two well-defined linear regions having different slopes (identified as Region-I and Region-II) in the ln­(*I*) vs ln­(*V*) plots for both devices indicates that the current transportation mechanism follows a power law behavior (*I* ∝ *V* ^m^).^38,70^ For both the cases, slopes are found to be ≈ 1 (i.e., *I* ∝ *V*) in the low forward voltage regions (i.e., Region–I), which signifies the Ohmic behavior and therefore termed as Ohmic region.^70^ However, slopes approaching a value 2 (i.e., *I* ∝ *V* ^2^) found in Region–II (i.e., higher forward voltage regime) for both devices (see Figure 14c-d) firmly suggest that the current flow through the metal–semiconductor junctions is governed by a trap-charge-free, space–charge-limited current (SCLC) conduction mechanism.^70,71^

All the device properties related to both the Schottky barrier diodes (SBDs) determined from the electrical measurements performed in dark conditions at room temperature have been tabulated in Table S6, Supporting Information.

The electrical conductivities of the present Fe­(III) and Co­(II) complexes were compared with representative literature-reported systems (Tables S7 and S8, respectively, Supporting Information). The Fe­(III) complex (complex **1**) exhibits conductivity higher than several Fe-based MOFs but lower than highly conductive mixed-valence iron compounds, indicating moderate semiconducting behavior [See Supporting Information, refs 20–23 ]. Similarly, the Co­(II) complex (complex **2**), shows conductivity comparable to several reported Co­(II) complexes and exceeds that of some low-conducting systems, although it remains lower than highly conductive cobalt coordination polymers and mixed-valence complexes [See Supporting Information, refs 24–30 ]. Overall, both complexes display semiconducting characteristics with conductivity values falling within the range commonly observed for transition-metal coordination compounds, highlighting their potential for SBD-based device applications. In spite of having favorable optical and electrical properties of the complexes for visible-spectral range optoelectronic applications, their practical realization remains impeded due to the challenges in preparing uniform, high-quality thin films.

### Theoretical Analysis for Conductivity

The electronic band structure has been plotted using a spin-resolved representation to account for the high-spin nature of Fe and Co complexes. In this scheme, the spin-up (α) and spin-down (β) channels are depicted in blue and red, respectively. The spin-resolved electronic band structure and projected density of states (PDOS) of the Fe-based system (C1) reveal a clear spin polarization, with distinct features for the α (red) and β (blue) channels. Both spin channels exhibit semiconducting behavior, although with different band gap magnitudes, the α channel displaying a wider gap than the β channel. Notably, several relatively flat bands appear within the band gap region, particularly in the energy range between approximately 2.0 and 4.5 eV, and are more pronounced in the α channel. These bands are indicative of localized electronic states, likely associated with Fe d-orbitals and their hybridization with ligand states (Figure 15, left).

**Figure 15:**
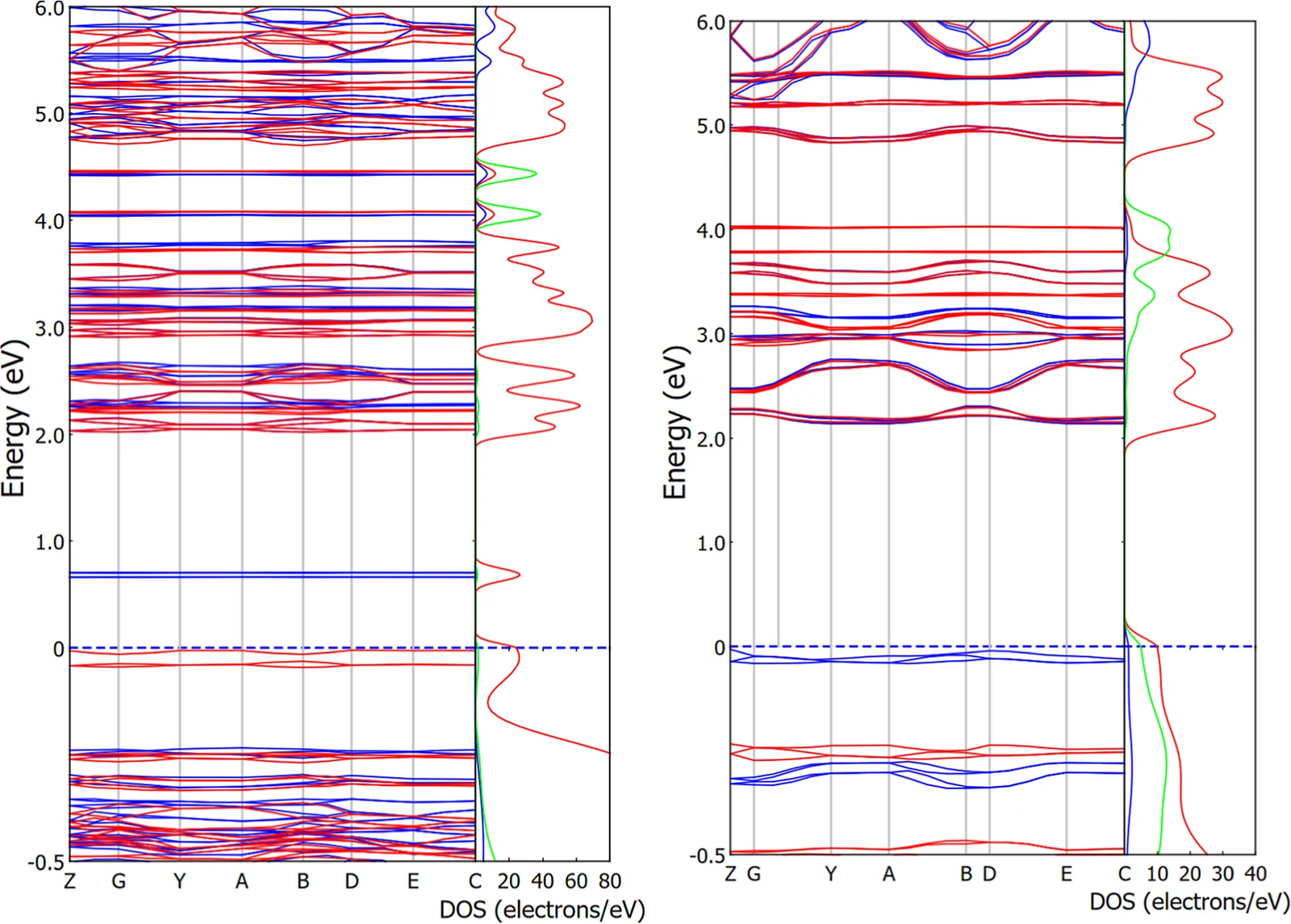
Spin-resolved electronic band structure and partial density of states (PDOS) of the C1 (left) and C2 (right) crystals. The α (spin-up) and β (spin-down) channels are represented in red and blue, respectively. High-symmetry points in the first Brillouin zone are labeled as follows: *Z* = (0, 0, 0.5), Γ = (0, 0, 0), *Y* = (0, 0.5, 0), *A* = (−0.5, 0.5, 0), *B* = (−0.5, 0, 0), *D* = (−0.5, 0, 0.5), *E* = (−0.5, 0.5, 0.5), and *C* = (0, 0.5, 0.5). PDOS is decomposed into s-states (blue), p-states (red), and d-states (green).

The PDOS further supports this interpretation, showing significant spin-polarized contributions in the vicinity of these intra-gap levels. In particular, the α channel exhibits a higher density of states in the conduction region, while the β channel shows a comparatively lower and more localized distribution near the Fermi level. The presence of these intermediate states within the band gap suggests additional charge transport pathways, such as thermally activated hopping or tunneling mechanisms, effectively reducing the transport gap.

In contrast, the Co-based system displays a well-defined spin-resolved electronic structure (Figure 15, right). As in the Fe system, the α channel shows a larger band gap than the β channel. However, no electronic states are observed within the band gap, indicating a clear separation between the valence and conduction bands. The bands near the Fermi level are relatively flat, suggesting a certain degree of electronic localization. However, the absence of intra-gap states points to a more conventional semiconducting behavior. The PDOS confirms this picture, showing a low density of states around the Fermi level for both spin channels, with no significant contributions within the gap region. The conduction band is mainly dominated by spin-polarized states, particularly in the α channel, whereas the β channel shows a more moderate contribution. To gain deeper insight into the origin of the electronic states, the projected density of states (PDOS) was decomposed into contributions from different atomic groups within the crystal, namely the organic ligands, the metal center, chloride ions, and coordinated water molecules (Figures 16 and 17).

**Figure 16:**
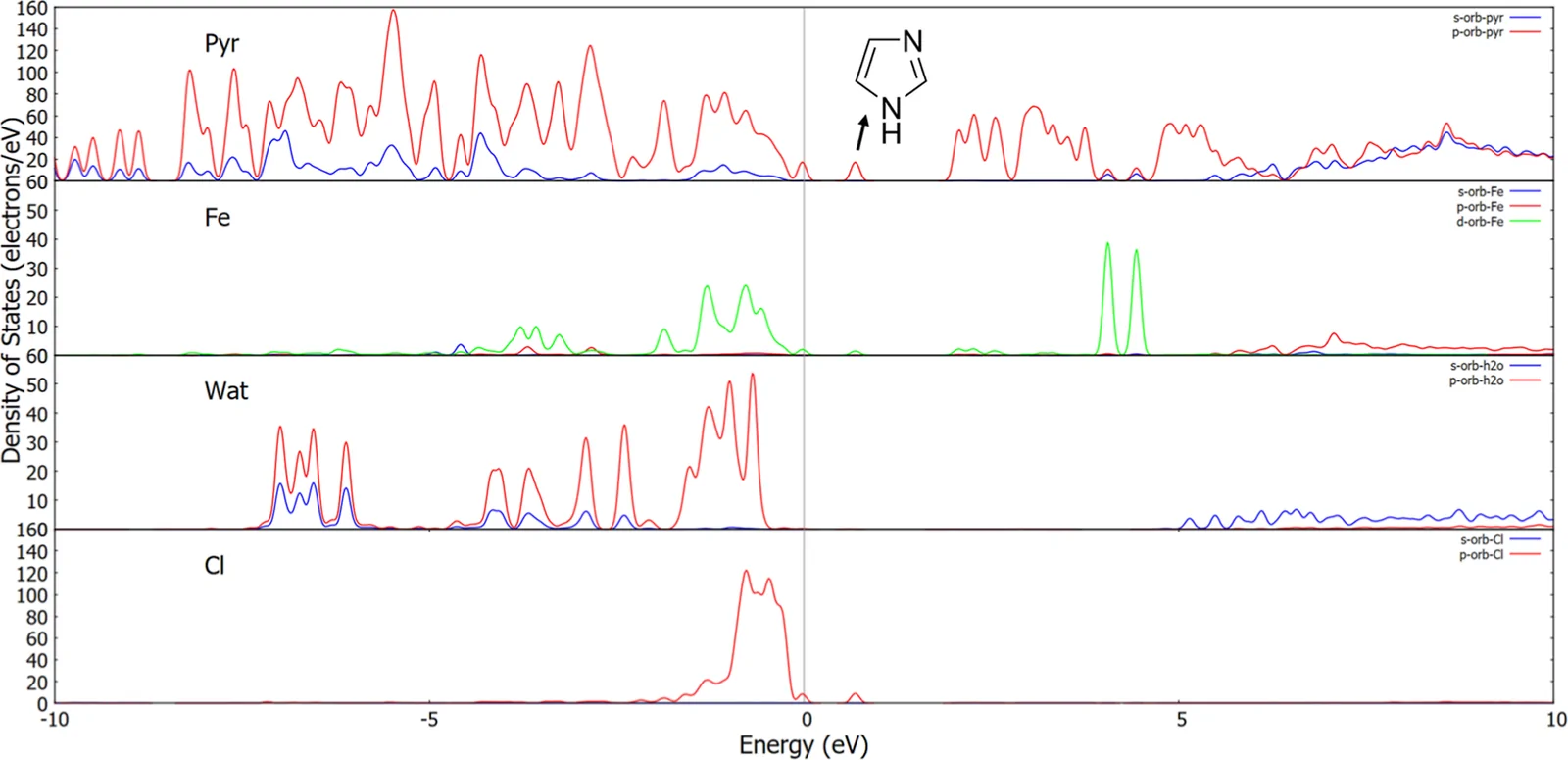
Calculated density of states of crystal cell **1**. The lines represent the s-orbital character (blue), p-orbital character (red), and d-orbital character (green) of the atoms into the crystal.

**Figure 17:**
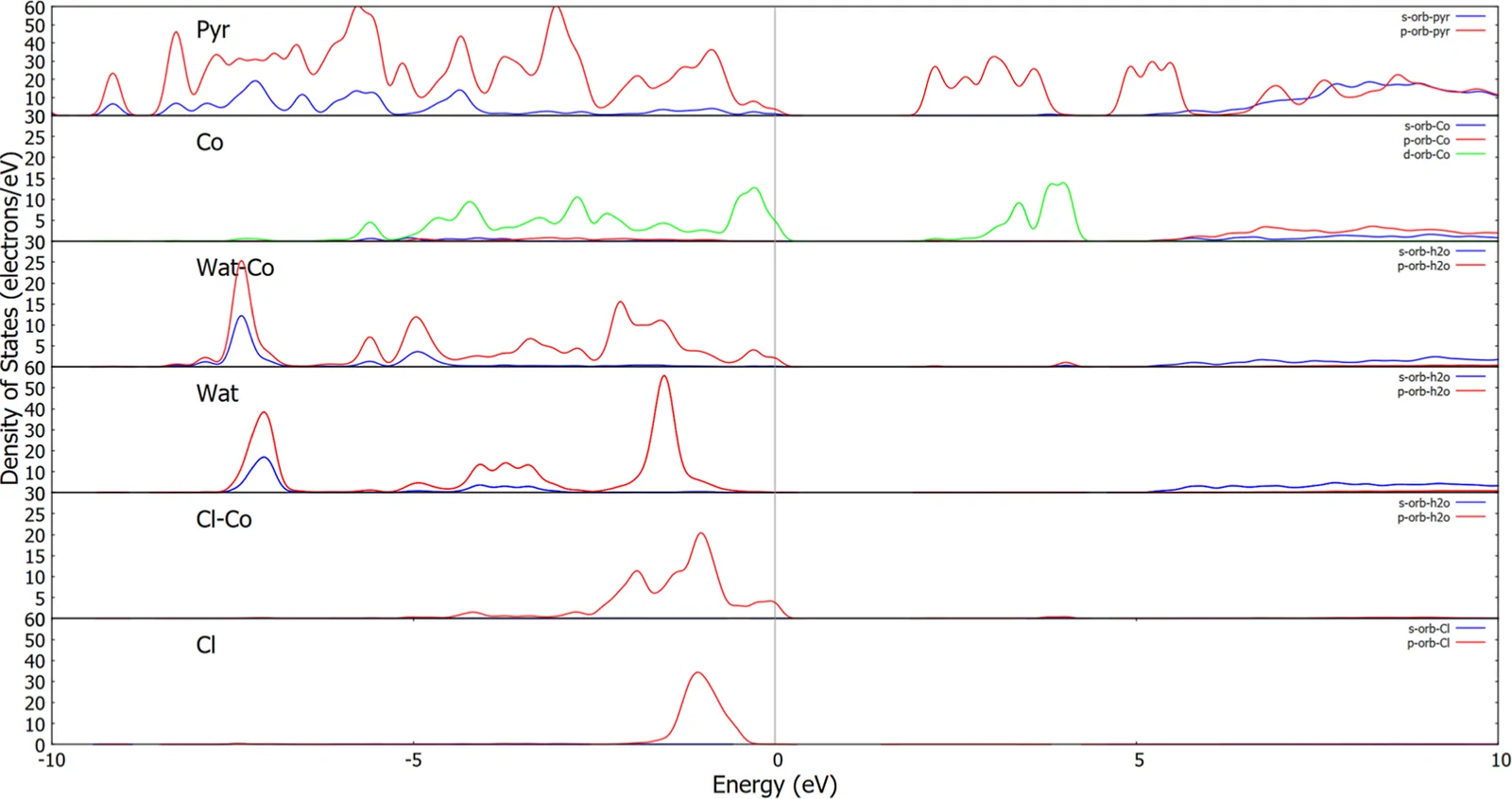
Calculated density of states of crystal cell **2**. The lines represent the s-orbital character (blue), p-orbital character (red), and d-orbital character (green) of the atoms into the crystal.

This decomposition allows the identification of the specific regions of the electronic structure associated with each component, enabling a clear assignment of the states forming the valence band, conduction band, and possible intra-gap levels. As a result, the spatial and chemical localization of the electronic states can be directly analyzed in terms of their atomic origins. The projected density of states (PDOS) shown in Figure 16 for the Fe-based complex **1** provides detailed insights into the orbital contributions governing its electronic structure. The valence band is mainly dominated by ligand-based p orbitals, particularly from the organic ligand, with minor hybridization from Fe d states. In contrast, the conduction band exhibits a stronger contribution from Fe d orbitals, indicating a partial metal-centered character of the unoccupied states.

A key feature of the electronic structure is the presence of intermediate states within the band gap. The PDOS clearly shows that these intra-gap states arise predominantly from the deprotonated imidazolyl fragment, with a smaller contribution from chloride ions and negligible involvement of Fe centers and coordinated water molecules.

The lack of significant Fe d character in these intermediate levels confirms their ligand-centered nature rather than metal-induced defect states. This localization highlights the important role of imidazolyl deprotonation in modulating the electronic structure by introducing accessible energy levels within the gap. As a result, these states can act as stepping stones for charge transport via hopping mechanisms, effectively reducing the transport gap and enhancing the electrical conductivity of the Fe-based systems.

In the Co-based system, both the chloride ions and water molecules play a dual role within the crystal structure. In addition to acting as coordinating ligands to the metal center, they are also present as counterions or crystallization species depending on their specific environment in the lattice.

In crystal **2**, the valence band is mainly dominated by p orbital contributions from the organic ligand, which define the occupied states near the Fermi level. Additional contributions from coordinated and crystallization water molecules appear in the deeper valence region, reflecting their localized character, while chloride contributes through p states slightly below the valence-band maximum (Figure 17).

The Co center contributes primarily via its d orbitals, which are distributed across the upper valence region and the onset of the conduction band. Importantly, the PDOS does not show any significant density of states within the band gap region, indicating a clean separation between the valence and conduction bands. This suggests that the coordination environment composed of the organic ligand, chloride, and water molecules does not induce localized intra-gap states, in contrast to the behavior observed in analogous Fe-based systems. As a result, the electronic structure of the Co complex is characteristic of a conventional semiconductor, where charge transport is expected to occur via direct band-to-band excitation rather than through hopping mechanisms mediated by localized states.

The dielectric response of the Fe-based system (complex **1**) exhibits a structured profile in both the real and imaginary components of the dielectric function (Figure S9, Supporting Information). In the low-energy region (<3 eV), the imaginary part shows multiple pronounced peaks, indicating the presence of several allowed electronic excitations, which can be associated with the intra-gap and near-edge states identified in the band structure and PDOS. These features are more intense and numerous than in the Co-based system, suggesting a richer distribution of accessible transitions at low energies. In the intermediate energy range (3–6 eV), the dielectric function of **1** displays broader and less intense features, consistent with transitions involving more delocalized states. At higher energies, both systems tend to converge toward similar dielectric behavior, indicating that the main differences are confined to the low-energy region. The optical conductivity spectra (Figure S9, Supporting Information) show that the Fe-based system (complex **1**) exhibits a slightly higher and more structured response in the low-energy region (<3 eV), consistent with the presence of additional electronic states facilitating low-energy transitions. In contrast, the Co-based system (**2**) displays a smoother onset, characteristic of a more conventional semiconductor. At higher energies, both systems follow similar trends, although **1** retains a more structured profile, reflecting its more complex electronic structure.

In summary, both systems exhibit semiconducting behavior with comparable band gap values and well-defined spin-resolved α and β channels. However, a key distinction arises in the Fe-based system, where intermediate states are present within the α channel, mainly associated with the deprotonated imidazole group. These intra-gap states, characterized by relatively flat bands, indicate localized electronic levels that may facilitate charge transport by providing additional pathways, such as thermally activated hopping or tunneling, effectively lowering the transport barrier. In contrast, the Co-based system lacks such intermediate states, and charge transport is therefore expected to occur via direct valence-to-conduction band excitation, resulting in a more conventional semiconducting behavior. It should be noted, however, that electrical conductivity in these materials may arise from multiple competing mechanisms, and thus, this interpretation should be considered with caution.

## Conclusion

In summary, we have successfully synthesized, structurally characterized, and evaluated the optoelectronic properties of two new iron­(III) and cobalt­(II) coordination complexes derived from the previously unexplored 4-imidazole-2,6-di­(pyrazinyl)­pyridine ligand. X-ray crystallography coupled with HS analysis demonstrates that both molecular lattices are stabilized by extensive 2D supramolecular networks governed by π···π stacking, classical/nonclassical hydrogen-bonding, and anion/lone-pair···π interactions. Theoretical modeling via MEP, QTAIM, and NCI plots confirms the critical role of these noncovalent contacts in maintaining crystal packing and structural integrity. Both complexes demonstrate suitable optical band gaps (2.02 eV for **1** and 2.16 eV for **2**) falling well within the visible regime. Fabricated ITO/sample/Au SBDs reveal clear rectifying behavior and space–charge-limited current (SCLC) conduction. Device **1** (Fe^III^) exhibits superior electrical performance, boasting a rectification ratio of ∼66 and an electrical conductivity nearly eight times higher than Device **2** (Co^II^). Periodic DFT calculations resolve the origin of this disparity, demonstrating that deprotonation of the imidazolyl fragment in complex **1** induces localized intra-gap states that serve as efficient intermediate pathways for charge hopping.

Looking forward, this work underscores the power of ligand modification and protonation-state control in tuning charge transport in 3d transition-metal complexes. A key future research direction will be expanding this structural design strategy to 4d and 5d transition metals. The broader spatial radial extension of 4d/5d orbitals is expected to increase metal–ligand electronic communication and intermolecular overlap, while stronger spin–orbit coupling could further modulate photophysical relaxation pathways and carrier lifetimes. Such comparisons between 3d and heavier 4d/5d analogues will provide deeper fundamental insights into designing cost-effective, high-performance molecular semiconductors for next-generation optoelectronic and energy-harvesting technologies.

## Supplementary Material







## Notes

Complex **1** (CIF)
Complex **2** (CIF)
Synthetic schemes; spectral data (FTIR); crystal data table; hydrogen-bonding parameters; geometrical parameters for π···π, anion···π interactions; supramolecular assemblies; theoretical methods for TD-DFT calculations; optical characterization; device fabrication; and device parameters and comparison tables (PDF)
