# Supplementary material for: From Molecular Crystals to Electronic Devices: Investigating Structure–Property Relationships of Iron(III) and Cobalt(II) Coordination Complexes
Source: J Phys Chem C Nanomater Interfaces. 2026 Sep 9;130(38):13363–80. doi: 10.1021/acs.jpcc.6c04146 (PMC13618249; doi:10.1021/acs.jpcc.6c04146)
Supplement: Supplementary file 1 [file jp6c04146_si_003.pdf]

SUPPORTING INFORMATIONS

From Molecular Crystals to Electronic Devices: Investigating Structure–Property Relationships of Iron(III) and Cobalt(II) Coordination Complexes

Subhajit Saha[a], Samit Pramanik[b], Sudipta Pathak[c], Riya Sadhukhan[d], Arnab Ghosh[e], Dipak K. Goswami[d], Hon Man Lee[f], Joaquín Ortega-Castro, Rosa M. Gomila, Antonio Frontera*[,g] and Subrata Mukhopadhyay*[,a]

[a]Department of Chemistry, Jadavpur University, Kolkata 700032, India

[b]Department of Basic Science and Humanities, Alipurduar Government Engineering and Management College, Bakla 736206, India

[c]Department of Chemistry, Haldia Government College, Debhog, PurbaMedinipur, West Bengal, 721657, India

[d]Organic Electronics Laboratory, Department of Physics, Indian Institute of Technology Kharagpur, Kharagpur 721302, India

[e]Department of Physics and Natural Science Research Centre of Belda College under Vidyasagar University, Belda, West Bengal 721424, India

[f]Department of Chemistry, National Changhua University of Education, Changhua 500, Taiwan 500

[g]Departament de Química, Universitat de les Illes Balears, Crta. de Valldemossa km 7.5, 07122, Palma de Mallorca (Baleares), Spain

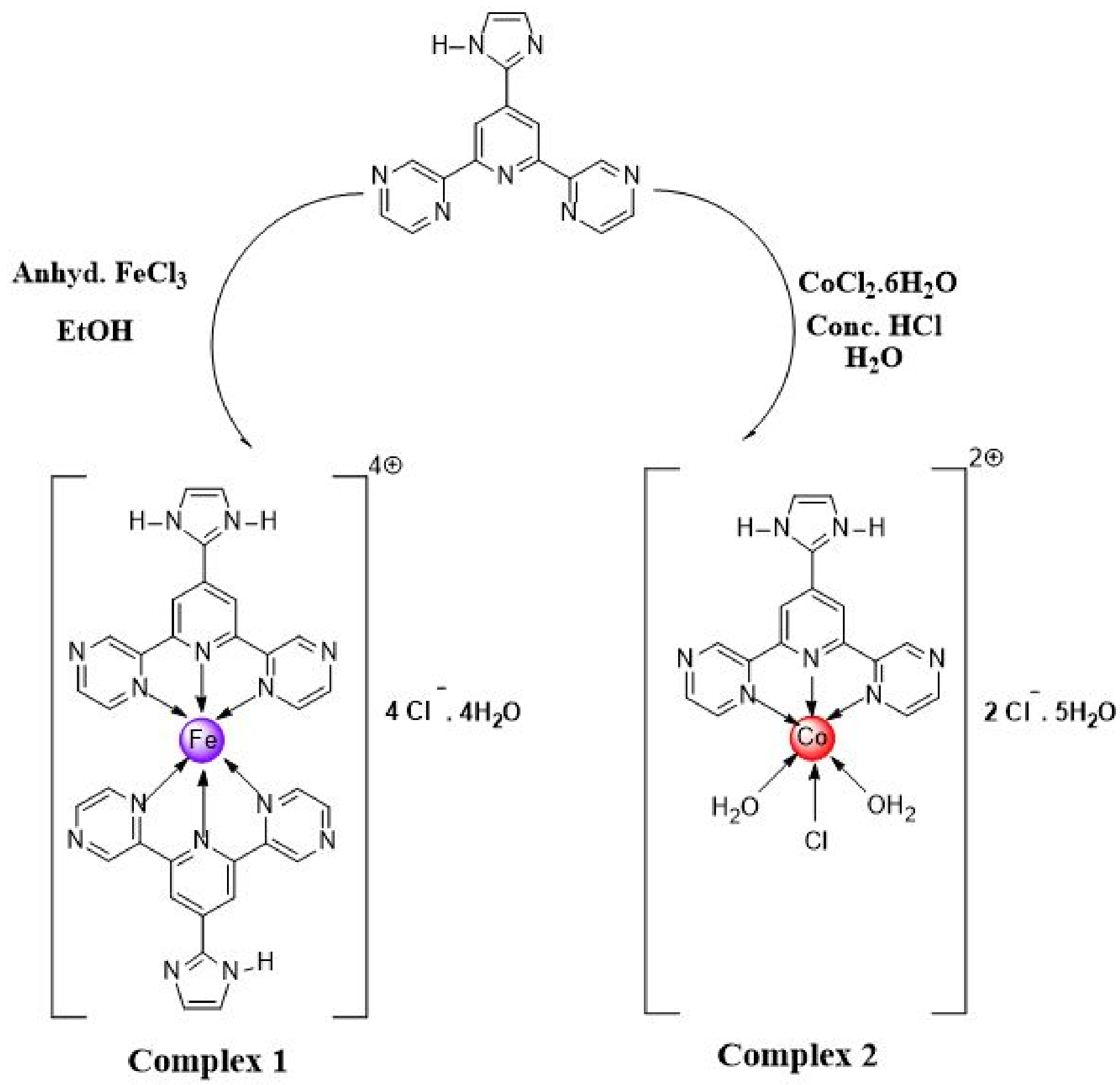

Scheme S1. Schematic Representation of the Synthesis of the Title Complexes

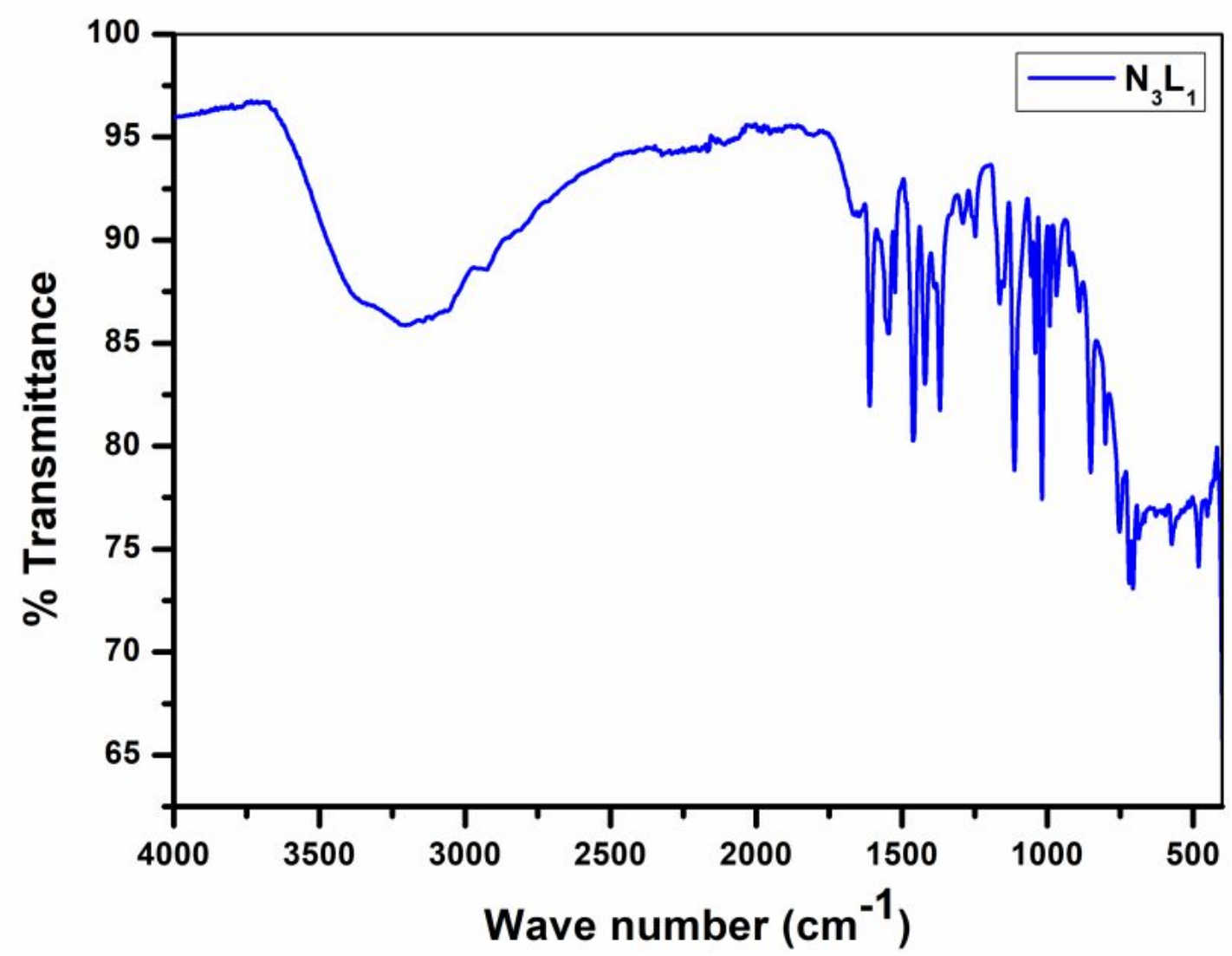

Figure S1. FT-IR spectrum of Ligand N_3L_1

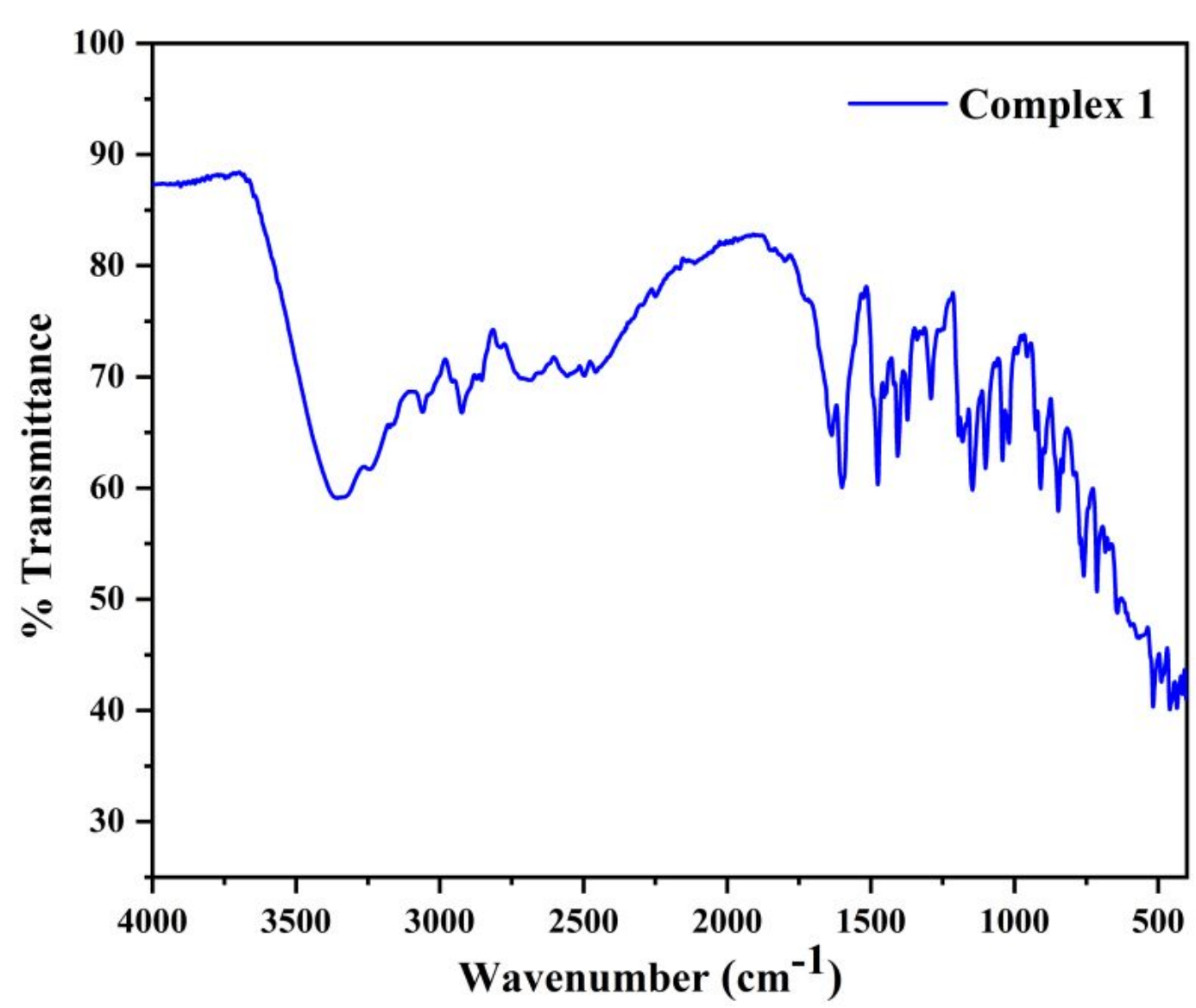

Figure S2. FT-IR spectrum of Complex **1**

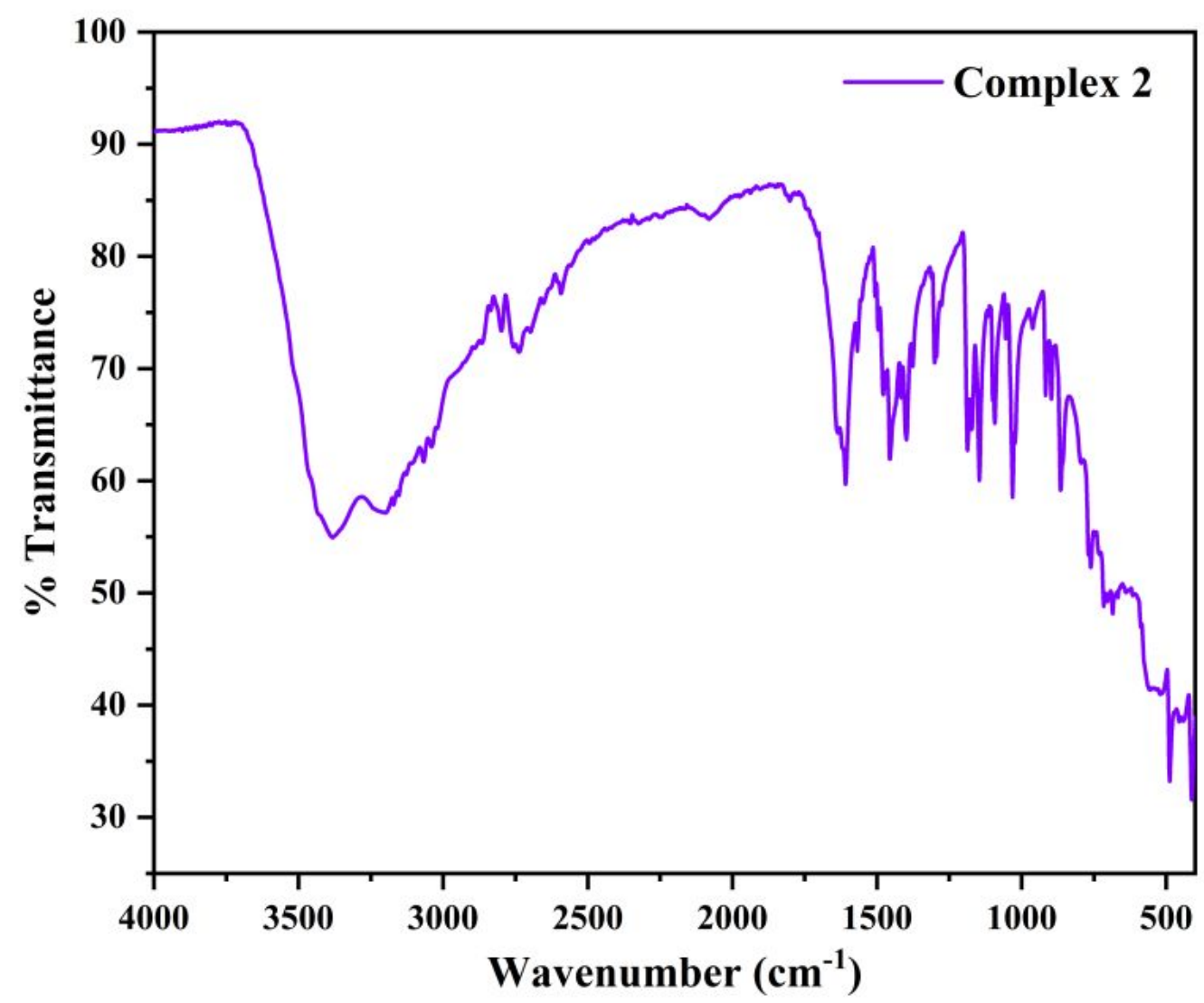

Figure S3. FT-IR spectrum of Complex **2**

Table S1. Crystal Data and Structure Refinement Parameters for Complexes 1 and 2.

Complex	1	2
Empirical formula	$C_{32}H_{31}N_{14}FeO_4Cl_4$	$C_{16}H_{22}N_7CoO_5Cl_3$
Formula Weight	873.50	557.68
Temperature (K)	298	300
Wavelength (Å)	Mo Kα (λ = 0.71073)	Mo Kα (λ = 0.71073)
Crystal system	Monoclinic	Monoclinic
space group	P 21/c	P 21/c
a, b, c (Å)	14.4048(5), 14.4177(5), 18.2551(7)	10.7362(2), 8.0552(5), 26.6890(16)
α, β, γ (°)	90, 99.525(1), 90	90, 93.688(2),90
Volume (Å^3)	3739.0(2)	2303.3(2)
Z / Density (calc.) (g/cm^3)	4/1.551	4/1.608
Absorption coefficient (mm^{-1})	0.531	1.136
F(000)	1792.871	1140
Crystal size (mm^3)	0.25 × 0.25 × 0.25	0.35 × 0.35 × 0.35
θ range for data collection	1.81 to 26.00	2.6 to 28.6
Completeness to θ (%)	99%	99%
Absorption correction	Multi-scan	Multi-scan

Max. and min. transmission	0.746 and 0.617	0.679 and 0.672
Refinement method	Full-matrix least-squares on F^2	Full-matrix least-squares on F^2
Data/Restraints/parameters	7341/0/493	5036/0/318
Goodness-of-fit on F^2	1.0966	1.109
Final R indices [I > 2σ(I)]	R_1 = 0.0464, wR_2 = 0.1198	R_1 = 0.0440, wR_2 = 0.1017
R indices (all data)	R_1 = 0.0664, wR_2 = 0.1198	R_1 = 0.0630, wR_2 = 0.1170
Largest diff. peak and hole (e.Å^{-3})	0.9035 and -0.8987	0.507 and -0.790

$R_1=\sum|| F_o|-|F_c||/\sum|F_o|$, $wR_2 = [\sum\{(F_o^2–F_c^2)^2\}/\sum\{w(F_o^2)^2\}]^{1/2}$ $w = 1/\{\sigma^2(F_o^2) + (aP)^2 + bP\}$; where, $P = (F_o^2 + 2F_c^2)/3$.

For Complex **1:** a = 0.0413 and b = 4.7811. For Complex **2:** a = 0.0412 and b = 4.010.

Table S2. Selected Bond Distances [Å] for Complex 1 and Complex 2

	**Bonds**	**Distance (Å)**	**Bonds**	**Distance (Å)**
Complex **1**	Fe1— N1	1.998(3)	Fe1—N4	1.977(2)
	Fe1—N2	1.888(2)	Fe1—N5	1.886(2)
	Fe1—N3	1.977(2)	Fe1—N6	1.990(2)
Complex **2**	Co1— Cl1	2.3455(10)	Co1—N1	2.183(2)
	Co1— O1	2.100(2)	Co1—N3	2.190(2)
	Co1— O2	2.120(2)	Co1—N5	2.075(2)

Table S3. Selected Bond Angles [°] for Complex 1 and Complex 2

	Bond angles	Value (°)	Bond angles	Value (°)
Complex **1**	N1—Fe1—N2	80.59(10)	N2—Fe1—N6	98.41(10)
	N1—Fe1—N3	161.13(10)	N3—Fe1—N4	92.00(9)
	N1—Fe1—N4	90.96(9)	N3—Fe1—N5	98.75(10)
	N1—Fe1—N5	100.12(10)	N3—Fe1—N6	91.35(9)
	N1—Fe1—N6	91.83(9)	N4—Fe1—N5	80.51(10)

	N2─Fe1─N3	80.55(10)	N4─Fe1─N6	161.19(10)
	N2─Fe1─N4	100.40(10)	N5─Fe1─N6	80.69(10)
	N2─Fe1─N5	178.86(10)		
Complex **2**	Cl1─Co1─ O1	89.31(8)	Cl1─Co1─ O2	90.18(7)
	Cl1─Co1─ N1	104.41(7)	Cl1─Co1─ N3	104.26(7)
	Cl1─Co1─ N5	178.72(7)	O1─Co1─ O2	175.58(11)
	O1─Co1─ N1	89.03(10)	O1─Co1─ N3	88.80(10)
	O1─Co1─ N5	91.95(10)	O2─Co1─ N1	86.84(9)
	O2─Co1─ N3	95.58(9)	O2─Co1─ N5	88.59(9)
	N1─Co1─ N3	151.22(9)	N1─Co1─ N5	75.89(9)
	N3─Co1─ N5	75.51(9)		

Table S4. Geometrical Parameters for the Hydrogen Bonds of Complex 1 and Complex 2

**D─H···A**	**D─H [Å]**	**H···A [Å]**	**D···A [Å]**	**D─H···A [°]**	**Symmetry**
**Complex 1**					
O1─H1B···Cl2	0.8500	2.240 (3)	3.095(3)	175.00	-
O2─H2A···O3	0.8500	2.065(4)	2.659(4)	126.00	-
O2─H2B···Cl2	0.8500	2.240(3)	3.220(16)	162.20	-
O3─H3A···Cl4	0.8510	2.616(4)	3.064(4)	114.00	-
N11─H11···Cl2	0.8600	2.208(3)	3.065(3)	173.90	2-x, 1-y, 1-z
N13─H13···Cl1	0.8600	2.208(3)	3.066(3)	174.80	1-x, 1-y, 1-z
N14─H14A···Cl3	0.8600	2.196(3)	3.024(3)	161.50	-
C2─H2···Cl3	0.9300	2.817(4)	3.520(3)	133.30	x, 1/2-y, -1/2+z
C7─H7···Cl4	0.9300	2.553(3)	3.471(3)	169.40	x, y, -1+z
C9─H9···Cl2	0.9300	2.669(3)	3.583(3)	167.80	2-x, 1-y, 1-z

C15─H15···Cl1	0.9300	2.609(3)	3.426(3)	146.90	
C22─H22···Cl2	0.9300	2.718(3)	3.626(3)	165.40	x, 1/2-y, -1/2+z
C29─H29···Cl1	0.9300	2.491(3)	3.415(3)	172.60	1-x, 1-y, 1-z
C40─H40···O4	0.9310	2.584(4)	3.167(4)	121.20	1-x, 1-y, 1-z
**Complex 2**					
O1─H1A···O3	0.76(5)	1.91(5)	2.662(6)	171.00	-
O2─H2B···O5	0.8200	1.8900	2.699(4)	168.00	1+x, y, z
O3─H3A···O4	0.8000	1.98(7)	2.777(7)	172.00(6)	x, 1+y, z
O3─H3B···O1	0.8300	2.54(8)	3.098(6)	126.00(7)	1-x, 1/2+y, 3/2-z
O2─H2A···N2	0.8100(5)	2.010(5)	2.812(3)	171.00(5)	x, -1+y, z
O1─H1B···Cl3	0.8200	2.2500	3.056(3)	168.00	-
O4─H4B···Cl3	0.8600	2.32(6)	3.168(4)	169.00(6)	1-x, -1/2+y, 3/2-z
N7─H7A···Cl3	0.8600	2.2700	3.130(3)	177.00	1-x, 1-y, 1-z
C12─H12···Cl3	0.9300	2.6000	3.519(3)	169.00	1-x, 1-y, 1-z
O3─H3B···Cl1	0.8300	2.59(9)	3.379(5)	159.00(8)	1-x, 1/2+y, 3/2-z
O4─H4A···Cl1	0.8100	2.43(6)	3.223(4)	167.00(5)	-
O5─H5A···Cl2	0.8500	2.3200	3.165(3)	171.00	-1+x, y, z
O5─H5B···Cl2	0.8500	2.3600	3.191(3)	164.00	1-x, 1-y, 1-z
N6─H6A···Cl2	0.8600	2.2800	3.128(3)	170.00	x, 1+y, z
C10─H10···Cl2	0.9300	2.5900	3.515(3)	171.00	x, 1+y, z

Table S5. Geometrical Parameters (Å, °) for the π-Stacking Interactions for the Title Complexes

**Cg(i)···Cg(j)**	**Cg(i)···Cg(j) [Å]**	**α (°)**	**β (°)**	**γ (°)**	**Cg(i)–perp[Å]**	**Cg(j)–perp[Å]**	**Symmetry**
**Complex 1**							

Cg(6)•••Cg(12)	3.9326(19)	1.05	28.00	27.88	3.476	3.472	1-x, 1-y, 1-z
Cg(12)•••Cg(6)	3.9327(19)	1.05	27.88	28.00	3.472	3.416	1-x, 1-y, 1-z
**Complex 2**							
Cg(3)•••Cg(5)	3.877(2)	7.13	26.47	33.60	3.230	3.471	1-x, 1-y, 1-z
Cg(5)•••Cg(3)	3.877(2)	7.13	33.60	26.47	3.471	3.230	1-x, 1-y, 1-z

Cg(i) and Cg(j) denotes centroid of ith and j^{th} ring respectively.

For Complex **1**: Cg(1) is the centroid of [Fe1/N1/C5/C6/N2] ring; Cg(3) is the centroid of [Fe1/N4/C25/C26/N5] ring; Cg(4) is the centroid of [Fe1/N5/C30/C31/N6] ring; Cg(6) is the centroid of [N13/C38/N14/C39/C40] ring; Cg(9) is the centroid of [N3/C11/C12/N9/C14/C15] ring; and Cg(12) is the centroid of [N6/C31/C32/N8/C34/C35] ring. For Complex **2**: Cg(3) is the centroid of [N6/C14/N7/C16/C15] ring and Cg(5) is the centroid of [N3/C5/C6/N4/C7/C8] ring.

X-ray crystallographic analysis

Single-crystal X-ray data were obtained using a Bruker SMART APEX II CCD area detector equipped with a graphite monochromated Mo Kα radiation (λ = 0.71073 Å) source in the φ and ω scan modes at room temperature for the complexes. The data acquisition and reduction were carried out using the CrysAlisPro 1.171.42.51a software.[1] Empirical absorption correction was performed using spherical harmonics implemented in SCALE3 ABSPACK.[1] The structures of both the complexes were solved by conventional direct methods and refined by full matrix least-squares methods using F^2 data. SHELXT and SHELXL 2018/3 programs were used for the solution and refinement of the structure of all the complexes respectively.[2] Final calculations were performed using Olex2 1.5.[3] CCDC 2552875 (for complex **1**) and 2552876 (for complex **2**) contain the supplementary crystallographic data for this work.

Theoretical Methods for Non-covalent Interactions

The interaction energies in the solid state were computed using the crystallographic coordinates at the PBE0-D3/def2-TZVP level of theory[4-6], optimizing only the positions of hydrogen atoms. All calculations were performed using the Gaussian-16 software package.[4] The PBE0 functional [1], a hybrid density functional that incorporates exact Hartree-Fock exchange, was chosen due to its reliable performance in describing electronic structures and noncovalent interactions. Grimme's D3 dispersion correction[5] was applied to accurately account for dispersion effects, which are particularly relevant for the noncovalent interactions analyzed in this study. The def2-TZVP basis set was used,[6] as it provides a well-balanced treatment of electron correlation effects and is widely adopted for transition metal and main-group element computations. Noncovalent interactions were characterized using both the quantum theory of atoms in molecules (QTAIM) method[7] and the non-covalent interaction (NCI) index with NCI plot isosurfaces.[8] QTAIM analysis provides a topological description of the electron density, enabling the identification of bond critical points (BCPs) and bond paths that define the nature of interactions. The NCI plot visualization complements this by offering a graphical representation of weak interactions based on the reduced density gradient (RDG). The isosurface color scheme follows a red-yellow-green-blue scale: red indicates strong repulsive interactions (ρ_{cut}^{+}), blue corresponds to strong attractive interactions (ρ_{cut}^{-}), and yellow and green denote weak repulsive and weak attractive interactions, respectively.[7]

Theoretical Methods for TD-DFT calculations

Excited-state properties were computed using TD-DFT as implemented in ORCA 6.1.[9] The CAM-B3LYP hybrid functional[10] combined with the D4 dispersion correction[11] (CAM-B3LYP-

D4) was employed in conjunction with the def2-TZVP basis set.[6] The CAM-B3LYP functional was selected for its superior performance in describing charge-transfer excitations and extended conjugated systems compared to conventional GGA and hybrid functionals. Solvent effects were incorporated using the Conductor-like Polarizable Continuum Model (CPCM)[12] with the appropriate solvent matching the experimental UV-Vis measurements: ethanol (ε = 24.9, n = 1.361) for Complex **1**, and water (ε = 78.36, n = 1.333) for Complex **2**. The CPCM cavity was constructed using van der Waals radii with a probe radius of 1.3 Å. A total of 50 excited states (singlet) were computed for each complex to comprehensively capture the electronic excitation landscape. Natural Transition Orbitals (NTOs)[13] were calculated to visualize and interpret the electron density redistributions associated with the most relevant electronic transitions. The calculated excitation energies, oscillator strengths, and orbital compositions were used to assign the experimentally observed absorption bands.

Theoretical Methods for Optical Study

Periodic density functional theory (DFT) calculations were performed using the plane-wave code CASTEP.[5] This approach was employed to investigate the structural and electronic properties of the studied crystalline systems. Geometry optimizations were carried out while keeping the lattice parameters fixed at their experimental values, allowing only atomic positions to relax.

The exchange–correlation interactions were described within the Generalized Gradient Approximation (GGA) using the Perdew–Burke–Ernzerhof (PBE) functional.[14,15] Long-range dispersion effects were included through the Grimme DFT-D3 correction scheme.[16] Ultrasoft pseudopotentials from the OTFG library were used, incorporating scalar relativistic effects following the Koelling–Harmon approach.[17] A plane-wave kinetic energy cutoff of 490 eV was

employed. The total energy convergence criterion was set to 2×10^{-6} eV. Brillouin zone sampling was performed using Monkhorst–Pack grids of 1 × 1 × 1 for C1 crystal and 1 × 2 × 1 for C2 crystal, which were found to be sufficient given the large unit cell sizes. To improve the description of localized d-electron states, single-point calculations were subsequently carried out within the DFT+U formalism.[18,19] Effective Hubbard U values of 5.0 eV for Fe and 3.3 eV for Co were applied. In the case of the Co-based system, both low-spin and high-spin configurations were considered. The high-spin state was found to be more stable, with an energy 0.521 eV lower than that of the low-spin configuration, confirming it as the ground state. Following the self-consistent field calculations, electronic band structures were computed along the standard high-symmetry k-path of the first Brillouin zone. The partial density of states total (PDOS) was evaluated and aligned with respect to the Fermi level. Optical properties, including the real and imaginary parts of the dielectric function as well as the optical conductivity, were calculated using a Gaussian smearing of 0.2 eV to simulate electronic transitions. For a better comparison with experimental data, a scissor operator of 0.20 eV was applied to correct the underestimated band gap.

Fabrication ITO/sample/Au Schottky devices:

For the fabrication of any efficient electronic or optoelectronic devices, coating a good uniform thin films of as-prepared material is mandatory because it bridges the properties of macro systems to molecular systems. For this work, to construct devices, first, homogeneous solutions (20 mg/ml concentration) of sample **1** and sample **2** were prepared in Dimethyl sulfoxide (DMSO) and deionized water (DI) water respectively. Schottky devices were fabricated on ITO-coated glass substrates (having sheet resistance ~15 Ω/cm^2, ITO thickness: 200 nm). The substrates underwent ultrasonic cleaning in acetone, 2-propanol (IPA), and deionized water (DI)

sequentially, each for a duration of 15 min. A hot plate was used for drying kept at around 80 °C. After that, 1 ml of each as- prepared solution was dispensed onto the cleaned ITO/glass substrates and spin-coated at speed of 1000 rpm, for one minute. Subsequently, the spin coated films were dried on hot plate, maintained at ~65 °C for 15 to get an average thickness of ≈1 ± 0.1 μm. The sandwiched Schottky device architectures were finalized by thermally evaporating top Au contacts, on the spin-coated thin film, through a 1.5 × 1.5 mm^2 square patterned shadow mask (effective device area $\approx 2.25 \times 10^{-6}$ m^2), at the base pressure of 6×10^{-6} mbar. Thickness of the spin-coated thin films were measured using Atomic Force Microscopy (AFM, Asylum Research). Hereafter, the Schottky devices prepared from sample **1** and sample **2** will be designated as device **1** and device **2** respectively.

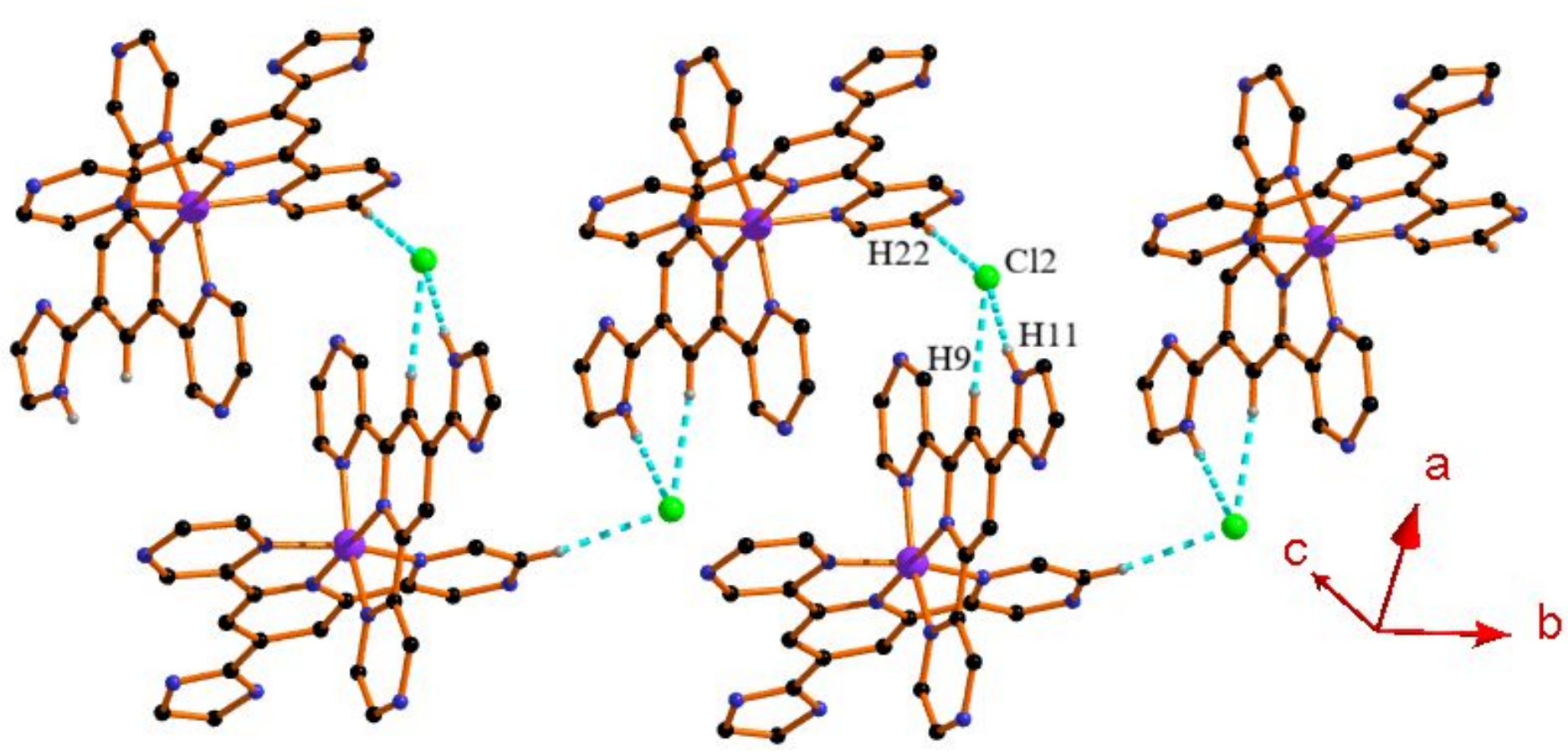

Figure S4. Formation of 1D zigzag polymeric chain incorporating C─H•••Cl and N─H•••Cl hydrogen bonding interaction in Complex **1**.

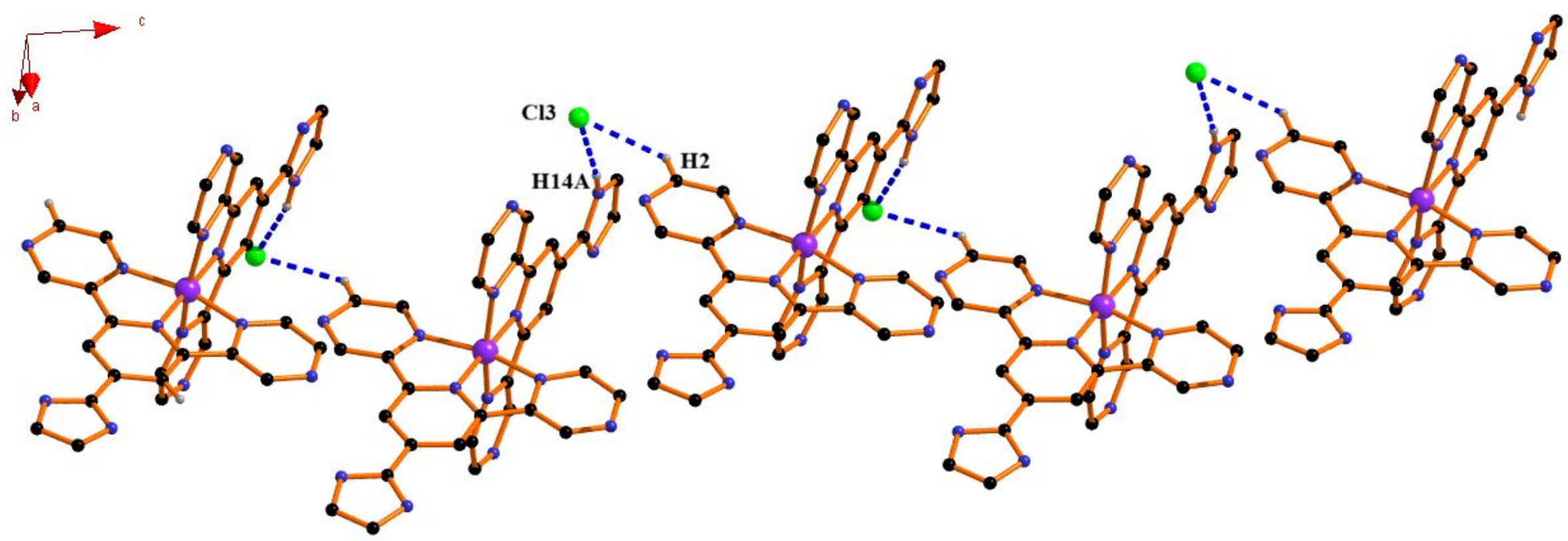

Figure S5. One dimensional (1D) polymeric chain formation via C─H•••Cl and N─H•••Cl hydrogen bonding in Complex **1**.

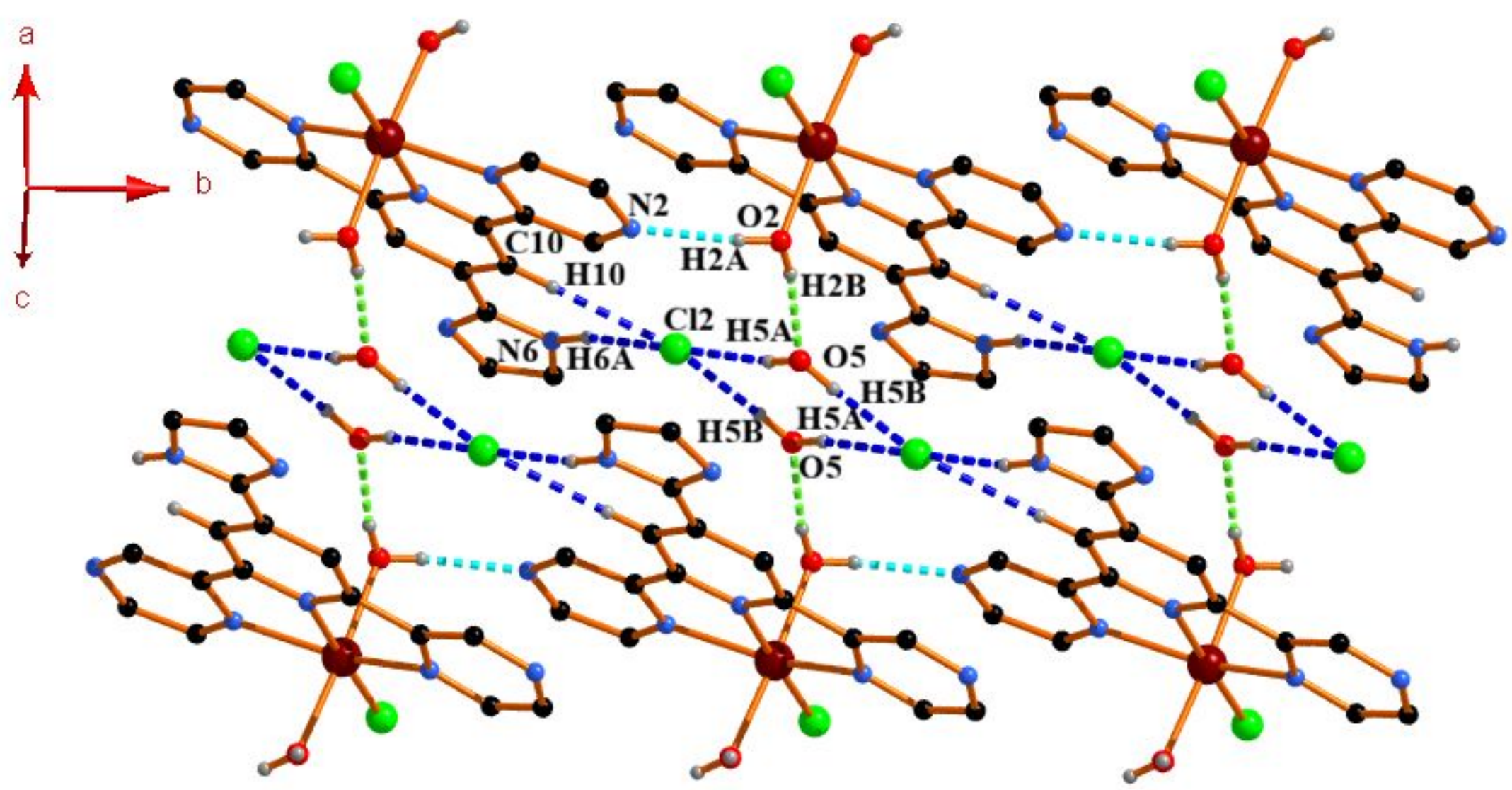

Figure S6. One-dimensional (1D) supramolecular tape formation via C─H•••Cl, N─H•••Cl and O─H•••Cl hydrogen bonding in Complex **2**.

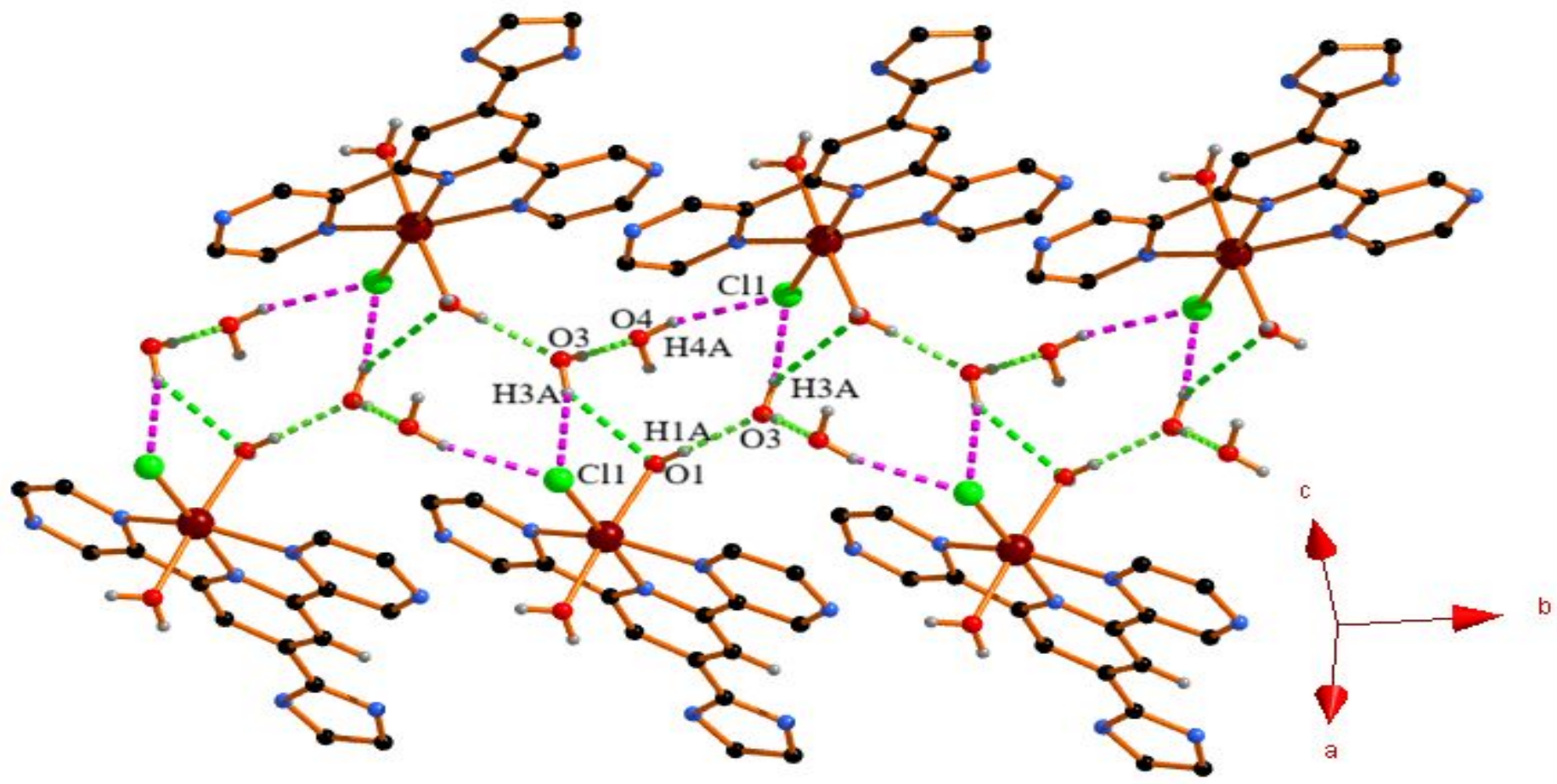

Figure S7. One dimensional (1D) supramolecular architecture formation via O─H•••Cl and O─H•••O hydrogen bonding in Complex **2**.

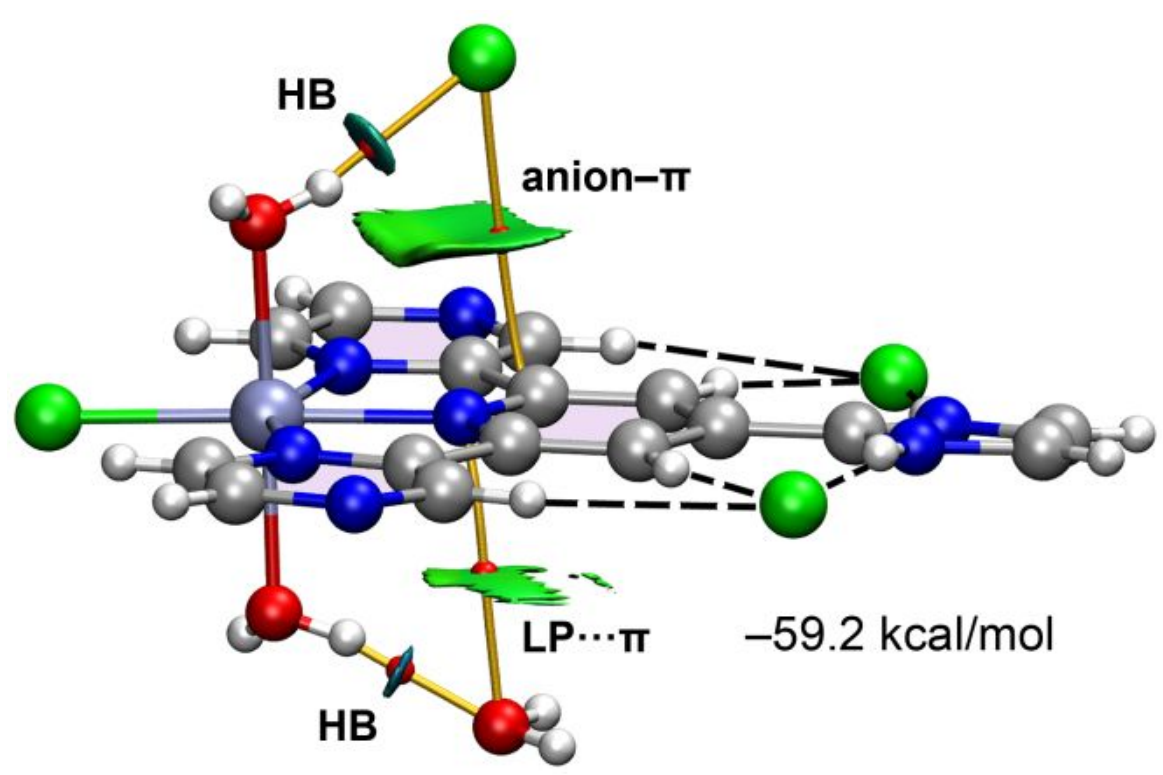

Figure S8. Combined QTAIM and NCI plot analysis for the anion•••π•••LP supramolecular assembly of Complex **2**. The green isosurfaces represent the reduced density gradient (RDG) surfaces, highlighting the regions of non-covalent interactions in real space.

Table S6: Extracted parameters of the Schottky diodes (Device **1** and Device **2**) measured at room temperature.

**Device**	**Rectification ratio (± 1 V)**	**Conductivity σ (S m^{-1})**	**Ideality factor (η)**	**Series Resistance R_s (kΩ)**

Device **1**	66	9.2×10^{-5}	4.1	1.2×10^3 (± 110)
Device **2**	17	1.14×10^{-5}	4.9	10.2×10^3 (± 150)

Table S7: Some representative conductivity values of Fe(II) and Fe(III) complexes with a comparison of the present work

Compound	Conductivity in $S.cm^{-1}$	Reference
$(NBu_4)_2Fe(III)_2(dhbq)_3$ (dhbq2−/3− = 2,5-dioxidobenzoquinone/1,2-dioxi do-4,5-semiquinone)	0.16 ± 0.01	20
$[Fe(C_{20}H_{23}N_3O_4)(N_3)]$	16.3×10^{-5}	21
$Fe(C_{22}H_{27}N_3O_4)(N_3)$	1.02×10^{-5}	
$Fe_2(DOBDC)(DMF)_2$ DOBDC = 2,5-dioxidobenzene-1,4-dicarboxylate	4.8×10^{-8}	22
$Fe_2(DSBDC)(DMF)_2$	5.8×10^{-7}	
$Fe_2(DSBDC)$ H_4DSBDC= 2,5-disulfhydrylbenzene-1,4-dicarboxylic acid	1.5×10^{-9}	
$Fe(1,2,3\text{-triazolate})_2(BF_4)_{0.33}$	7.7×10^{-5}	23
$C_{32}H_{31}N_{14}FeO_4Cl_4$	9.2×10^{-7}	Present Work

Table S8: Some representative conductivity values of Co(II) complexes with a comparison of the present work

Compound	Conductivity in $S.cm^{-1}$	References
$C_{38}H_{50}Co_3N_{16}O_5.CH_3OH$	1.05×10^{-8}	24
$C_{46}H_{65.33}Co_3N_4O_{14.67}$	5.858×10^{-6}	25

$C_{58.04}H_{69.59}C_{11.59}Co_3N_4O_{16.78}S_{1.59}$	6.804×10^{-7}	
$C_{62.50}H_{77.50}C_{11.50}Co_3N_4O_{16.50}S_{1.50}$	9.358×10^{-7}	
$C_{28}H_{32}Co_2N_9O_8$	2.04×10^{-7}	26
$C_{30}H_{36}Co_2N_4O_9S,0.33H_2O$	2.04×10^{-7}	27
$C_{26}H_{22}CoN_2O_6$	2.61×10^{-7}	28
$C_{26}H_{20}Br_2CoN_2O_7$	2.52×10^{-8}	
$C_{34}H_{40}N_8O_{12}Co$	5.76×10^{-6}	29
$C_{48}H_{38}CoN_2O_6$	7.66×10^{-7}	30
$C_{16}H_{22}N_7CoO_5Cl_3$	1.14×10^{-7}	Present Work

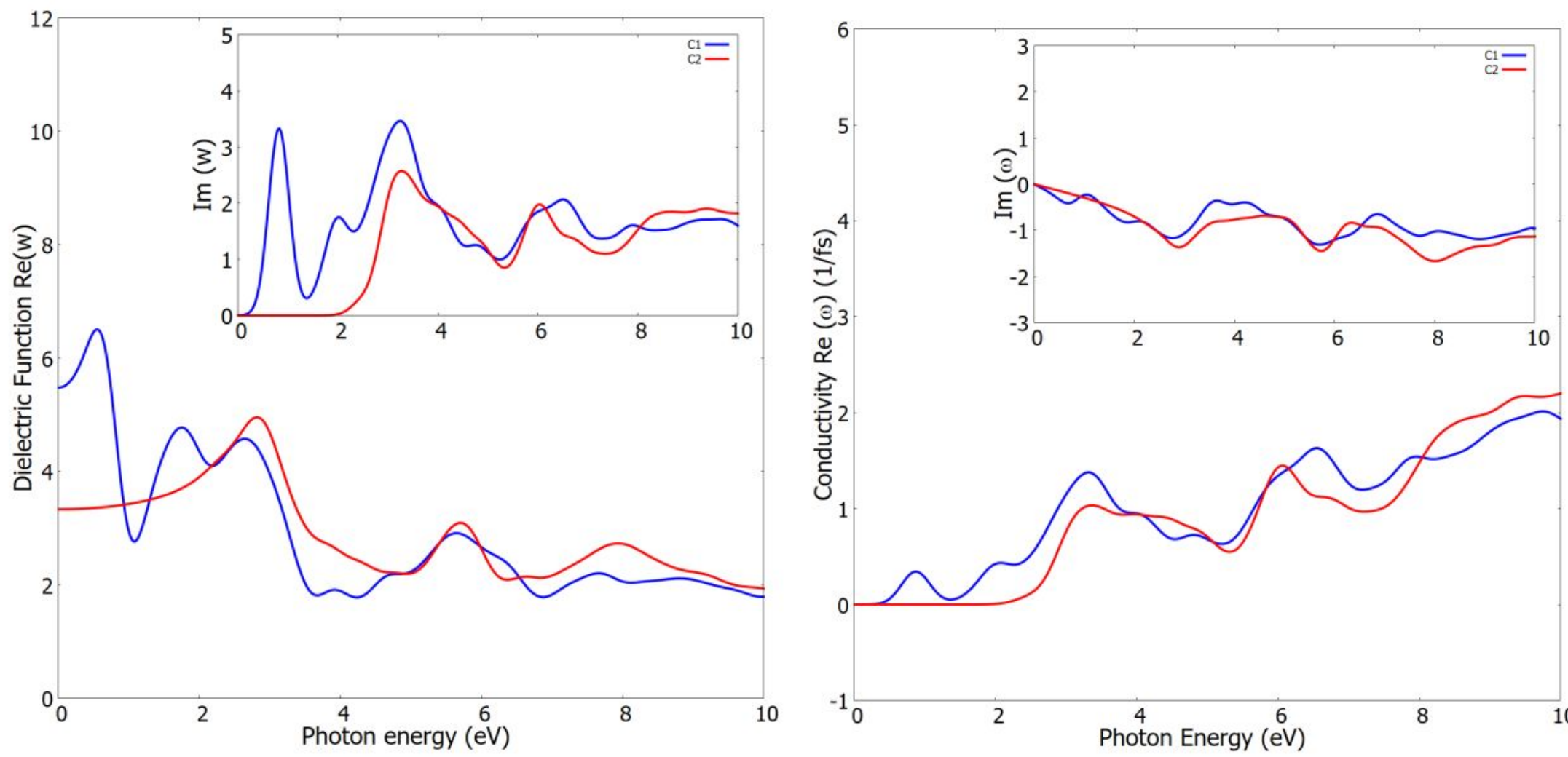

Figure S9. Real and imaginary dielectric and conductivity function of **1**(blue lines) and **2**(red lines) over all polarization directions.

References:

(1) Rigaku. *CrysAlisPro 1.171.42.51a*; Rigaku Oxford Diffraction: **2022**

(2) Sheldrick, G. M. *SHELXL*; University of Göttingen, Germany: **2015**.

(3) Dolomanov, O. V.; Bourhis, L. J.; Gildea, R. J.; Howard, J. A. K.; Puschmann, H. "OLEX2: a complete structure solution, refinement and analysis program". *J. Appl. Cryst.* **2009**, *42*, 339- 341.

(4) Grimme, S.; Antony, J.; Ehrlich, S.; Krieg, H. A Consistent and Accurate. *J. Chem. Phys.* **2010**, *132*, 15404.

(5) Weigend, F.; Ahlrichs, R. Balanced Basis Sets of Split Valence, Triple Zeta Valence and Quadruple Zeta Valence Quality for H to Rn: Design and Assessment of Accuracy. *Phys. Chem. Chem. Phys.* **2005**, *7* (18), 3297–3305.

(6) Adamo, C.; Barone, V. Toward Reliable Density Functional Methods without Adjustable Parameters: The PBE0 Model. *J. Chem. Phys.* **1999**, *110* (13), 6158–6170.

(7) Contreras-garcía, J.; Johnson, E. R.; Keinan, S.; Chaudret, R.; Piquemal, J.; Beratan, D. N.; Yang, W. NCIPLOT : A Program for Plotting Noncovalent Interaction Regions. *J. Chem. Theory Comput.* **2011**, *7* (3), 625–632.

(8) Rose, W.; Clark, S. J.; Ii, M. D. S.; Ii, C. J. P.; Iii, P. J. H.; Iv, M. I. J. P.; V, K. R. Universities of Leeds , Sheffield and York First Principles Methods Using CASTEP. *Z. Kristallogr. Cryst. Mater.* **2005**, 567–570.

(9) Neese, F. Software update: the ORCA program system, version 5.0. *Wiley Interdisciplinary Reviews: Computational Molecular Science* **2022**, *12*(5), e1606.

(10) Kumar, J.; Nakashima, T.; Kawai, T. Circularly Polarized Luminescence in Chiral Molecules and Supramolecular Assemblies. *J. Chem. Phys. Lett.* **2015**, *6*(17), 3445−3452.

(11) Caldeweyher, E.; Ehlert, S.; Hansen, A.; Neugebauer, H.; Spicher, S.; Bannwarth, C.; Grimme, S. A Generally Applicable Atomic-Charge Dependent London Dispersion Correction. *J. Chem. Phys.* **2019,** *150*, 154122.

(12) Tomasi, J.; Mennucci, B.; Cammi, R. Quantum Mechanical Continuum Solvation Models. *Chem. Rev.* **2005**, *105*(8), 2999–3094.

(13) Martin, R. L. Natural Transition Orbitals. *J. Chem. Phys.* **2003**, *118*(11), 4775–4777.

(14) Perdew, J. P.; Burke, K.; Ernzerhof, M. Generalized Gradient Approximation Made Simple. *Phys. Rev. Lett*. **1996**, *77* (18), 3865–3868.

(15) Perdew, J. P.; Jackson, K. A.; Pederson, M. R.; Singh, D. J.; Fiolhais, C. Atoms, Molecules, Solids, and Surfaces: Applications of The Generalized Gradient Approximation For Exchange and Correlation. *Phys. Rev. B.* **1992**, *46* (11), 6671–6687.

(16) Grimme, S.; Chemie, T. O.; Münster, O. I. D. U. Semiempirical GGA-Type Density Functional Constructed with a Long-Range Dispersion Correction. **2006**, *27* (15), 1787–1799.

(17) State, S.; You, P. A Technique for Relativistic Spin-Polarised Calculations. *J. Phys. C: Solid State Phys.* **1977**, *10* (16), 3107–3114.

(18) Cococcioni, M.; Gironcoli, S. De. Linear Response Approach to the Calculation of the Effective Interaction Parameters in the LDA+ U Method. *Phys. Rev. B - Condens. Matter Mater. Phys.* **2005**, *71* (3), 1–16.

(19) Kresse, G.; Blaha, P. Comment on “ Taming Multiple Valency with Density Functionals : A Case Study of Defective Ceria.” *Phys. Rev. B - Condens. Matter Mater. Phys.* **2005**, *72* (23), 237101(1–2).

(20) Darago, L. E.; Aubrey, M. L.; Yu, C. J.; Gonzalez, M. I.; Long, J. R. Electronic Conductivity, Ferrimagnetic Ordering, and Reductive Insertion Mediated by Organic Mixed-Valence in a Ferric Semiquinoid Metal-Organic Framework. *J. Am. Chem. Soc.* **2015**, *137* (50), 15703–15711.

(21) Basak, T.; Das, D.; Ray, P. P.; Banerjee, S.; Chattopadhyay, S. Two Iron (III) Based Semiconductors with Theoretical. *CrystEngComm*. **2020**, *22* (31), 5170–5181.

(22) Sun, L.; Hendon, C. H.; Minier, M. A.; Walsh, A.; Dincə, M. Million-Fold Electrical Conductivity Enhancement in Fe2(DEBDC) versus Mn2(DEBDC) (E = S, O). *J. Am. Chem. Soc.* **2015**, *137* (19), 6164–6167.

(23) Organic, M. M.; Fe, F.; Park, J. G.; Aubrey, M. L.; Oktawiec, J.; Chakarawet, K.; Darago, L. E.; Grandjean, F.; Long, G. J.; Long, R. Charge Delocalization and Bulk Electronic Conductivity in the Mixed-Valence Metal−Organic Framework Fe(1,2,3-Triazolate)2(BF4)X. *J. Am. Chem. Soc.* **2018**, *140* (27), 8526–8534.

(24) Bhunia, S.; Das, M.; Banerjee, S.; Drew, M. G. B.; Ray, P. P.; Chattopadhyay, S. Application of a Distinctly Bent, Trinuclear, End-to-End Azide Bridged, Mixed Valence Cobalt(III/II/III) Complex in the Fabrication of Photosensitive Schottky Barrier Diodes. *RSC Adv.* **2024**, *14* (16), 11185–11196.

(25) Bhunia, S.; Das, P.; Banerjee, S.; Gomila, R. M.; Drew, M. G. B.; Frontera, A.; Ray, P. P.; Chattopadhyay, S. Fabrication of Schottky Barrier Diodes Utilizing Carboxylate Bridged Trinuclear Mixed Valence Cobalt(III/II/III) Complexes of Tetradentate N2O2 Donor Reduced Schiff Base Ligands. *New J. Chem.* **2023**, *47* (30), 14202–14216.

(26) Banerjee, A.; Das, D.; Ray, P. P.; Banerjee, S.; Chattopadhyay, S. Phenoxo-Bridged Dinuclear Mixed Valence Cobalt(III/II) Complexes with Reduced Schiff Base Ligands:

Synthesis, Characterization, Band Gap Measurements and Fabrication of Schottky Barrier Diodes. *Dalton Trans.* **2021**, *50* (5), 1721–1732.

(27) Sarkar, R. S.; Biswas, A.; Ray, P.; Gomila, R. M.; Drew, M. G. B.; Banerjee, S.; Frontera, A.; Chattopadhyay, S. An Insight into the Non-Covalent Interactions in the Solid State Structures of Dinuclear Cobalt (II) Complexes with N , O-Donor Ligands : Application of the Complexes in the Fabrication of Schottky Devices. *CrystEngComm*. **2023**, *25* (6), 1006–1017.

(28) Ahmed, F.; Datta, J.; Khan, S.; Dutta, B.; Islam, S.; Naaz, S.; Ray, P. P.; Mir, M. H. Linear Dicarboxylate-Based Pyridyl-Appended Cobalt(II) Coordination Polymers in Search of Opto-Electronic Properties. *New J. Chem.* **2020**, *44* (21), 9004–9009.

(29) Das, P.; Maiti, A.; Ghosh, S.; Ray, P. P.; Ghoshal, D. Exploring the Dual Realm of Solvent-Responsive Structural Transformations and Semiconducting Properties of Six 1D Coordination Polymers. *Cryst. Growth Des*. **2024**, *24* (14), 6017–6027.

(30) Hossain, E.; Sk, R.; Das, M.; Ray, P. P.; Frontera, A.; Mir, M. H.; Mukhopadhyay, S. Schottky Device Fabrication of Linear Dicarboxylato-Bridged Mn(II) and Co(II) Coordination Polymers: Experimental and Theoretical Insights. *Cryst. Growth Des.* **2024**, *24* (18), 75978–7604.
